# The Origin of Large Molecules in Primordial Autocatalytic Reaction Networks

**DOI:** 10.1371/journal.pone.0029546

**Published:** 2012-01-04

**Authors:** Varun Giri, Sanjay Jain

**Affiliations:** 1 Department of Physics and Astrophysics, University of Delhi, Delhi, India; 2 Jawaharlal Nehru Centre for Advanced Scientific Research, Bangalore, India; 3 Santa Fe Institute, Santa Fe, New Mexico, United States of America; Umeå University, Sweden

## Abstract

Large molecules such as proteins and nucleic acids are crucial for life, yet their primordial origin remains a major puzzle. The production of large molecules, as we know it today, requires good catalysts, and the only good catalysts we know that can accomplish this task consist of large molecules. Thus the origin of large molecules is a chicken and egg problem in chemistry. Here we present a mechanism, based on autocatalytic sets (ACSs), that is a possible solution to this problem. We discuss a mathematical model describing the population dynamics of molecules in a stylized but prebiotically plausible chemistry. Large molecules can be produced in this chemistry by the coalescing of smaller ones, with the smallest molecules, the ‘food set’, being buffered. Some of the reactions can be catalyzed by molecules within the chemistry with varying catalytic strengths. Normally the concentrations of large molecules in such a scenario are very small, diminishing exponentially with their size. ACSs, if present in the catalytic network, can focus the resources of the system into a sparse set of molecules. ACSs can produce a bistability in the population dynamics and, in particular, steady states wherein the ACS molecules dominate the population. However to reach these steady states from initial conditions that contain only the food set typically requires very large catalytic strengths, growing exponentially with the size of the catalyst molecule. We present a solution to this problem by studying ‘nested ACSs’, a structure in which a small ACS is connected to a larger one and reinforces it. We show that when the network contains a cascade of nested ACSs with the catalytic strengths of molecules increasing gradually with their size (e.g., as a power law), a sparse subset of molecules including some very large molecules can come to dominate the system.

## Introduction

One of the puzzles in the origin of life is the question: How did large molecules, which are essential for all cells to function, first arise? Macromolecules such as RNA and protein molecules, which contain from about a hundred to several thousand monomers, are produced in cells with the help of two crucial catalysts (a) the RNA polymerase which reads the genes on DNA molecules and produces the corresponding messenger RNA molecules and (b) the ribosome which reads the messenger RNA molecules and produces the corresponding protein molecules. These two powerful catalysts, RNA polymerase and ribosome, are themselves made up of proteins and RNA molecules, each of which is produced by the process mentioned above. When cells produce daughter cells, the latter are already endowed with these catalysts at birth, from which they synthesize other molecules. Nowhere in the living world is there a natural process we know of that produces macromolecules and that does *not* itself use macromolecules. Hence the puzzle. We expect that the answer to the question lies in the processes that occurred before life originated.

The Miller experiment [1] and subsequent work [2]–[5] were successful in synthesizing monomer building blocks of large molecules in simulated prebiotic environments. Those experiments suggested that amino acids and nucleotides, monomer building blocks of macromolecules, could be produced on the prebiotic earth. Subsequently there has been much experimental work to explore mechanisms that could enhance the concentrations of monomers and synthesize long polymers [6]–[9]. While there is interesting progress, as yet there is no compelling scenario for the primordial origin of large molecules.

Meanwhile what has been observed is that catalysis is a fairly ubiquitous property that arises in different kinds of molecules and even at small sizes. Organocatalysts [10]–[12], peptides [13], [14], and RNA molecules [15]–[17] are known to have catalytic properties. Cofactors play an important role in catalyzing metabolic reactions and they (or their evolutionary predecessors) may have had a role in prebiotic catalysis [18].

The ubiquity of catalysis motivates the main idea behind the present paper. Here we attempt to investigate theoretically, using a mathematical model, whether one can construct a chemical organization that produces large molecules from small ones, using the property of catalysis. Apart from the specific question of the origin of large molecules the present work is also motivated by a larger question of how complex structures and organizations are built incrementally from simpler ones. In systems where catalysis is possible an important self-organizing structure that can appear is an autocatalytic set (ACS). ACSs were proposed by Eigen [19], Kauffman [20] and Rossler [21] and have been used by many authors to study various aspects of self-organization, evolution and the origin of metabolism [22]–[31], the origin of replication [32]–[34], and the origin and dynamics of protocells [35]–[38]. In order to separate the issues, the present model only has catalysis and no replication or spatial enclosures; we wish to see what can be achieved by catalysis alone.

Farmer et al [22], Bagley et al [39], and Bagley and Farmer [23] proposed and analyzed a model of an artificial chemistry in which polymers could form by ligation of shorter polymers through spontaneous reactions as well as reactions catalyzed by other polymers in the chemistry. Bagley and Farmer [23] analyzed the population dynamics of the molecular species and established some important properties of autocatalytic self-organization. When the food set (monomers) were supplied at a fixed input rate and the chemistry contained an ACS they showed that in a suitable range of parameters the concentrations of the ACS molecules dominated over the rest of the molecules (the background), thereby focusing the chemical resources of the system into a small subset of molecules comprising the ACS. However the largest polymers in the ACSs they considered had about 15–20 monomers; they did not systematically investigate the problems that arise in generating much larger molecules in their chemistry.

These problems were sharply articulated in the work of Ohtsuki and Nowak [34], in which they considered a much simpler model that could be analytically solved. In this model, which they refer to as ‘symmetric prelife’ with a catalyst, they showed that in order for the catalyst to acquire a significant concentration in a prebiotic scenario its catalytic strength should be very large, growing exponentially with its length. The inference from the model, therefore, was that it is difficult for a large catalyst molecule to arise in a prebiotic scenario.

In this work we consider a model of artificial chemistry similar in structure to that of Bagley and Farmer. This model is intermediate in complexity and realism between the model of Bagley and Farmer (which is slightly more complex) and model of Ohtsuki and Nowak (which is much simpler). We study the dynamics of this model in the presence of ACSs and in particular a structure that we refer to as a ‘nested ACS’ in which a small ACS helps trigger a larger one. We show that this mechanism when iterated across a cascade of nested ACSs avoids the problem of exponentially growing catalyst strengths. This mechanism, therefore, provides a possible route to the construction of large molecules in a pre-biotic scenario. Apart from these results our work provides an insight, based on the analytic treatment of the system under certain approximations as well as numerical work, of certain ACS properties and questions such as why ACSs dominate, why nested ACSs work, etc.

## Results

### The Model

The model is specified by describing the set of molecular species, their reactions, and the dynamical rate equations for their population dynamics. A special set of molecules, the ‘food set’, denoted 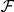, consists of small molecular species, 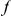 in number, that are presumed to be abundantly present in a prebiotic niche. The simplest version of the model (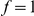) contains only a single monomer species A (or A(1)) whose concentration 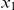 in a well stirred prebiotic region will be assumed to be buffered (constant). The other molecules, A(2), A(3),… (dimers, trimers, etc.), whose concentrations are denoted 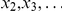, are all made through ligation and cleavage reactions of the type 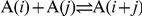 with forward (ligation) rate constant denoted 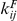 and reverse (cleavage) rate constant 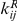. The net forward flux of this reaction pair is given by 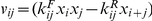. The rate equations for the system are given by 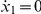, and, for 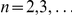,
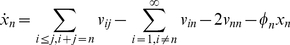
(1)

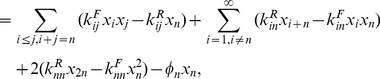
(2)where 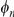 represents a loss rate of species 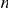 from the region in question. The two terms in the first sum represent the formation (respectively, cleavage) of 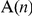 from (into) smaller molecules. The two terms in the second sum and the following bracket represent the cleavage (respectively, formation) of larger molecules via reactions that produce (consume) 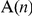. The stoichiometric factor of 2 before the bracket arises because two molecules of 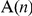 are involved in the corresponding reaction pair. The set of parameters 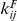, 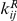 that are non-zero define the set of possible reactions; collectively they define the ‘spontaneous chemistry’ (‘spontaneous’ in the sense that the reactions are possible even in the absence of catalysts). A pair of ligation and cleavage reactions can be excluded from the chemistry by setting both 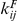 and 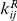 to zero. The scheme permits chemistries in which some reactions proceed in only one direction (ligation or cleavage) by setting only one of 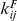 and 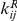 to zero. However, we will primarily be interested in a chemistry in which each reaction is reversible. The existence of the cleavage reactions makes it more difficult for the long molecules to survive; thus it is more significant to demonstrate the appearance of long molecules in a model in which cleavage reactions are permitted than in one where only the forward (ligation) reactions are.

We consider a simple scheme for catalyzed reactions, assuming that a molecule enhances the rate of a reaction that it catalyzes in proportion to its own concentration. Thus, if 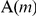 is a catalyst of the reaction pair 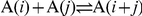, then the rate constants of this reaction pair, 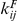 and 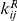, are replaced 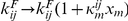 and 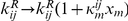, where 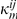 is the ‘catalytic strength’ of the catalyst for this reaction pair. The first term in the bracket, unity, represents the spontaneous reaction rate (which is present irrespective of whether the reaction is catalyzed or not), and the second term 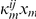 represents the enhancement of the reaction rate due to the catalyst. Note that in this scheme a catalyst enhances both the forward and reverse reaction rates by the same factor. If a reaction has multiple catalysts, 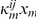 is replaced by 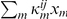, where the sum runs over all catalysts 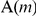 of the reaction in question. Typically, only a small subset of the spontaneous reactions will be catalyzed. The set of catalyzed reactions together with the catalysts and their catalytic strengths will be referred to as the ‘catalyzed chemistry’.

When there are 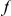 food set (or ‘monomer’) species a general molecule A is represented as an 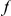-tuple of non-negative integers: 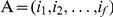, where 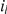 is number of monomers of type 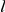 contained in A. The identity of a molecule in the model is completely determined by the number of monomers of each type contained in the molecule; the order in which they appear is irrelevant. Thus the combinatorial diversity of distinct compounds containing a total of 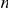 monomers (of all types) grows only as a power of 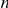 (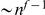) instead of exponentially (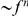 for strings) if the order had mattered. This simplification helps in picturizing the chemistry and significantly reducing the computational power needed to explore large values of 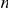. The reaction scheme and rate equations are similar to the ‘1-dimensional’ version above. Details of the general model and explicit examples of rate equations for 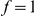 and 2 are discussed in the Appendix S1.

The main differences between the present model and that of Bagley and Farmer are (a) a simpler representation of molecules (we do not consider molecules as strings), (b) a simpler treatment of catalysis (we do not consider intermediate complexes), and (c) we ignore the effects coming from small populations containing a discrete number of molecules. We reproduce the main phenomenon of ACS dominance that Bagley and Farmer observed, but the relative simplicity of the present model allows us to explore other phenomena that they do not report about (this includes a multistability in the dynamics and the possibility of building large molecules through nested ACSs).

The main differences with the model of Ohtsuki and Nowak are (a) a much richer spontaneous chemistry of ligation reactions and the inclusion of reverse reactions (which makes an analytical treatment more difficult), and (b) a much more general class of catalyzed chemistries, instead of a single catalyst (which allows us to talk of nested ACSs, in particular). With a specific choice of parameters our 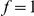 model reduces exactly to their ‘symmetric prelife’ model with a catalyst. In spite of greater complexity we are able to numerically reproduce their main results in a much more general setting, and also provide approximate analytical understanding of the results.

#### Autocatalytic Sets (ACSs)

The dynamics of the above system is particularly interesting when ACSs are present in the catalyzed chemistry. Consider a set 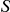 of catalyzed one-way reactions. ‘One-way’ means that each reaction in 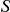 is either a ligation or cleavage reaction. Thus the set of reactants and the set of products are unambiguously defined for each reaction and the two sets are distinguished. The presence of a given ligation or cleavage reaction in 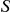 does not mean that its reverse is also necessarily a member of 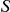. Let 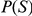 be the union of sets of products of all reactions in 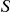, and 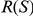 the union of sets of reactants of all reactions in 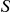. We exclude the food set molecules from both 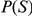 and 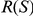. We will refer to the set 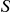 of catalyzed reactions as an ACS if (a) 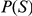 includes a catalyst for every reaction in 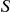, and (b) 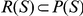. The latter condition implies that all members of 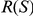 can be produced from the food set by (recursively) applying reactions from within 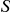. An ACS thus ensures the existence of a catalyzed pathway, starting from the food set, for the production of each of its products [19]–[21]. Alternative valid definitions of an ACS can be given (see [40], [41] for one such); the above definition suffices for our present purposes and we hope to return to consequences of other kinds of ACSs in the future. Note that if 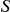 is an ACS, then its extension, 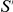, that additionally includes the reverse of some reactions in 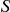, is also trivially an ACS, as in our scheme a catalyst works for both forward and reverse reactions if both exist in the chemistry.

### Spontaneous (uncatalyzed) chemistries

#### Nomenclature

We first consider the case when none of the reactions is catalyzed, 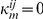 for all 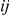 pairs. For concreteness we first consider spontaneous chemistries that are ‘reversible’, ‘homogeneous’ and ‘fully connected’. A ‘reversible’ chemistry is one for which each allowed reaction is reversible, i.e., 
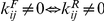
. A ‘homogeneous’ chemistry is one in which all the nonzero rate constants are independent of the species labels: 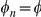 for all 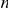, 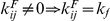 independent of 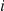 and 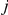, and 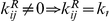 independent of 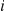 and 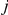. A ‘fully connected’ chemistry is one in which all possible ligation and cleavage reactions are allowed: 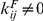 and 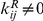 for all 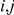. A chemistry is ‘connected’ if every molecule can be produced from the food set in some pathway consisting of a sequence of allowed reactions. In this paper we discuss spontaneous chemistries that are reversible and homogeneous. We have checked that introducing irreversible reactions and bringing in a small amount of heterogeneity does not change the conclusions. Some results for sparse chemistries are discussed later. For homogeneous and fully connected chemistries the model has 4 parameters, 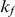, 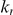, 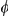, and the concentration of the monomer, 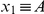.

We explore the model numerically and, to a limited extent, analytically. While the chemistry under consideration is infinite, numerical simulations were done by choosing a finite number 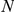 for the size of the largest molecule in the simulation. In simulating Eq. (1) all terms corresponding to reactions in which any molecule larger than 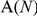 is produced or consumed were omitted. In principle this introduces another parameter, 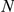, an artifact of the simulation. However, one expects that most properties of physical interest should become independent of 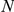 when 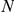 is sufficiently large. Evidence for this is presented in Appendix S2. Our numerical solution of differential equations was mostly done using the CVODE solver library of the SUNDIALS (Suite of Nonlinear and Differential/Algebraic Equation Solvers) package [42], and, for smaller 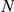 values, using XPPAUT [43]. Steady states obtained were verified using numerical root finders in Octave [44] and Mathematica [45].

#### Steady state properties of the spontaneous chemistry: Populations decline exponentially with the size of molecules

**Figure 1:**
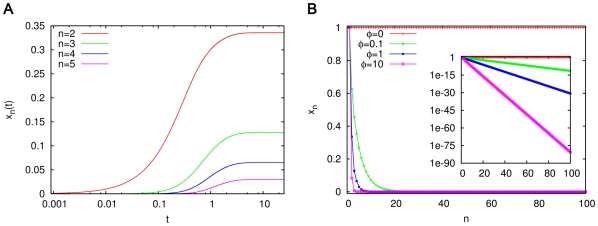
Concentrations in uncatalyzed chemistries with a single food source. (A) Evolution of concentrations with time for a chemistry with 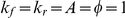. For simulation purposes, the size of the largest molecule was taken to be 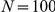. (B) Steady state concentration as a function of molecule size. Parameters take the same values as in (A) except that four values of 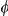 are shown, 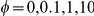. Inset shows the same on a semi-log plot; the straight lines are evidence of exponential damping of 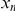 for large 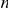 (Eq. (3)), with 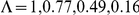 for the four cases, respectively. 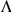 is computed from the slope of a straight line fit after ignoring the smaller molecules (up to 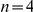 in this case).

Starting from the initial condition in which all concentrations other than the food set are zero (we refer to this as the standard initial condition), the concentrations were found to increase monotonically and reach a steady state (Fig. 1A). Numerically the graph of steady state 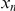 versus 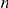 on a semi-log plot was found to be approximately a straight line for large 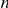, consistent with the expression
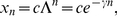
(3)where, 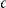 and 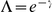 are constants. 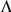, determined by numerically fitting the slope, decreases monotonically as 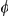 increases (Fig. 1B).

For 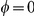, the following exact analytical solution for the steady state concentrations exists for homogeneous and connected uncatalyzed chemistries:
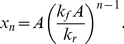
(4)To see that this is a fixed point, note that when Eq. (4) holds, then 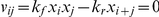 for all 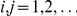; hence the r.h.s. of Eq. (1) vanishes (at 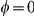). Thus 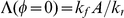. Hence, whenever 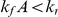, the steady state concentrations of large molecules are exponentially damped, 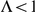.

When 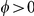 we do not have an analytic solution. Numerically, we find that 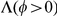 drops to below 1 even when 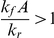. 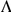 is found to be a monotonically increasing function of 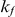 and 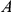, and a monotonically decreasing function of 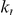 and 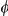. This corresponds to the intuition that an increased ligation rate favours large molecules and an increased cleavage or dissipation rate disfavours them. By casting the rate equation in terms of dimensionless variables one can easily see that there are only two independent parameters, which may be taken to be 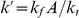 and 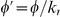 whenever 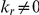 (for details see Appendix S3). Alternatively when 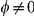, we can take the two dimensionless parameters to be 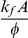 and 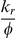. The dependence of 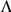 on these two sets of parameters is also shown in Appendix S3. The uncatalyzed chemistry seems to have a global fixed point attractor (all initial conditions tested lead to the same steady state).

**Figure 2:**
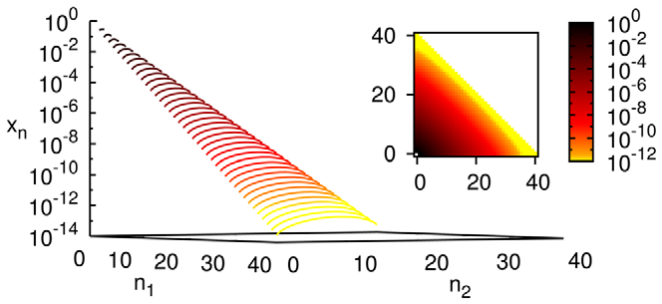
Steady state concentration profile in an uncatalyzed chemistry with 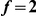. The 3D plot shows the concentration 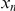 of the molecule 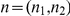 as a function of 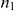 and 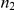 in the steady state, for an uncatalyzed chemistry with 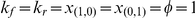, 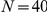. The inset shows a ‘top view’ of the (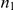,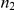) plane with 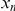 indicated in a colour map on a logarithmic scale.

Similar results hold when two food sources are present in the system (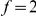) with buffered concentrations of the monomers (1,0) and (0,1). Simulations are done with all possible reaction and cleavage reactions allowed between molecules containing a maximum of 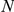 monomers, all with the same forward rate constant 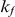 and reverse rate constant 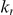 and a common dissipation rate 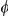 for the molecules. A steady state concentration profile is shown in Fig. 2. ‘Diagonal entries’ (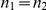) have higher concentrations in homogeneous chemistries because there are more reaction pathways to build molecules with equal numbers of both monomers than unequal. Since the number of species goes as 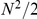 and the number of reactions as 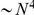, computational limitations require us to work with a smaller 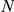 than for 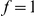. Qualitative conclusions nevertheless appear to be 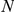 independent.

### Chemistries with autocatalytic sets

#### ACS molecules dominate the population in certain parameter regions

**Figure 3:**
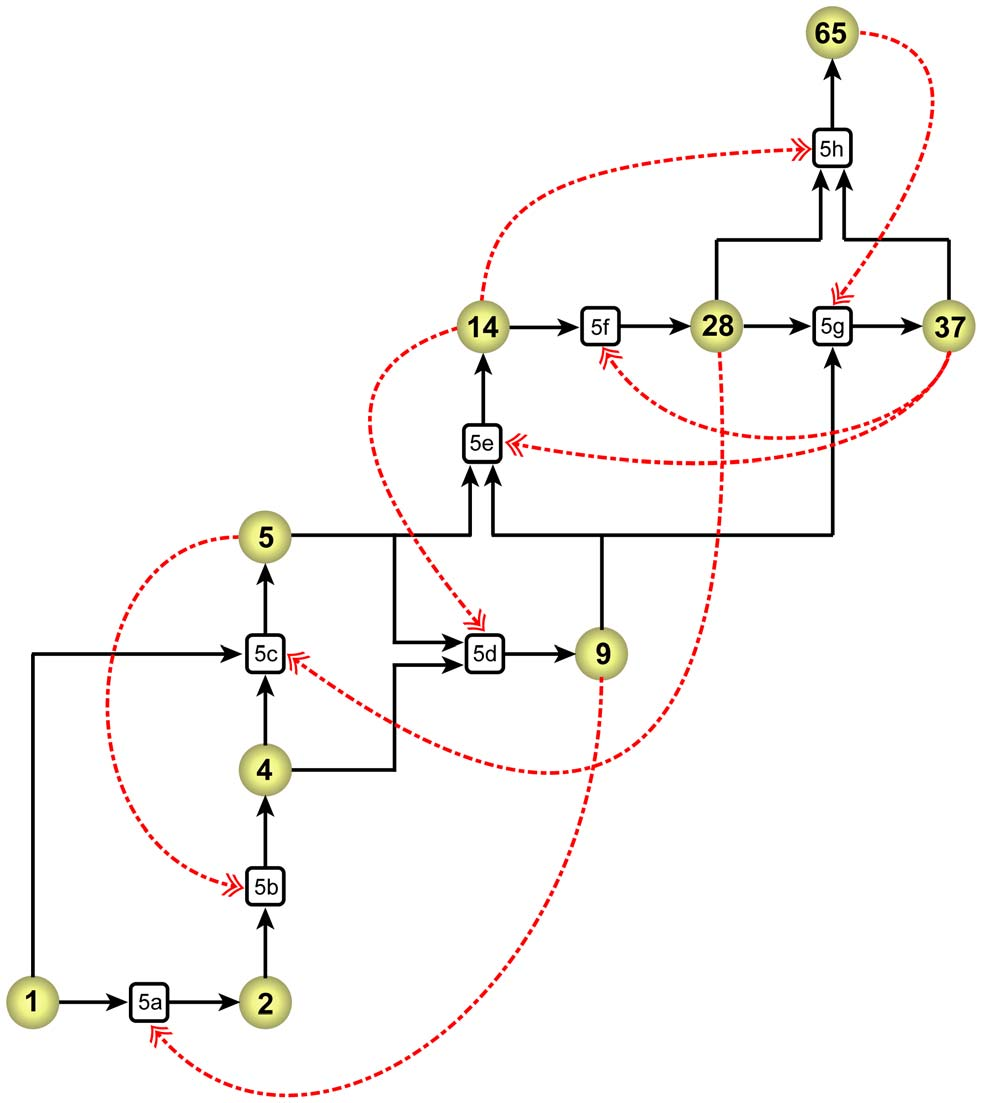
Pictorial representation of the catalyzed chemistry in **Eqs. (5)**, referred to as ACS65. This is a directed bipartite graph with two types of links. Circular nodes represent molecules and rectangular nodes represent reactions. The numbers inside the nodes identify the nodes (molecule size 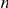 for circular nodes and reaction equation number for rectangular nodes). A black solid arrow from a molecule to a reaction node indicates that the former is a reactant in the latter, and one from a reaction to a molecule node that the latter is a product of the former. A red dashed arrow from a molecule to a reaction node indicates that the former is a catalyst for the latter. To avoid visual clutter some black arrows starting from molecule nodes are shown to branch out into more than one arrow. (For example, the arrow from molecule node 5 branches into reaction nodes 5d and 5e; this means that molecule 5 is a reactant in both reactions. This structure should not be construed as a bi-directional link between reaction nodes 5d and 5e.) The figure only represents the ligation reactions in the catalyzed chemistry; the reverse (cleavage) reactions are not shown.

We now consider chemistries which contain some catalyzed reactions in addition to the spontaneous reactions described above. As a specific example to display certain generic properties, we consider the catalyzed chemistry defined by equations (5) below and represented pictorially in Fig. 3:
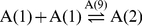
(5a)

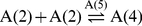
(5b)

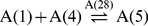
(5c)

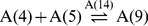
(5d)

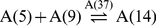
(5e)

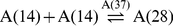
(5f)

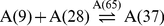
(5g)

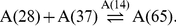
(5h)


**Figure 4:**
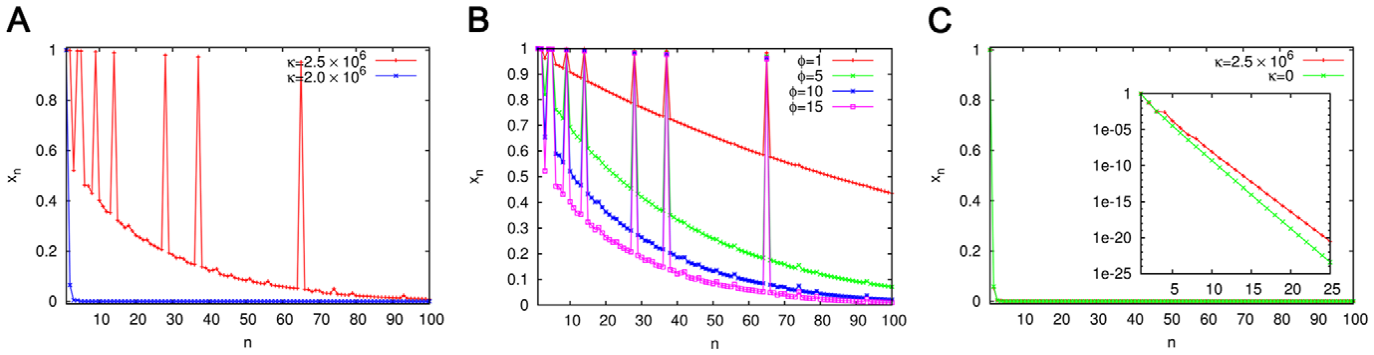
Steady state concentration profile for ACS65 (**Eqs. (5)**). In all the cases 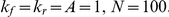 (A) The concentration profile for two values of 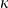 for 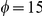. (B) The concentration profile for four values of 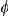 for 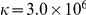. (C) The concentration profile for 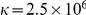, 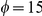 but with reaction (5a) removed from the ACS (red curve) compared with the profile for the spontaneous chemistry, 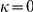, 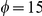 (green curve). The inset shows the same with 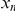 on a logarithmic scale. On the linear scale the two curves are indistinguishable.

Note that this set of reactions constitutes an ACS (which we will refer to as ACS65). If any one reaction pair is deleted from the set, it is no longer an ACS. For the moment, for simplicity, we consider the case where the catalytic strengths of all the catalyzed reactions are equal (‘homogeneous’ catalytic strengths): 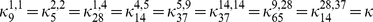, and all other 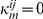. (For clarity, in view of double digit indices, we have introduced a comma between the pair of indices in the superscript.) Fig. 4A describes the steady state concentrations, starting from the standard initial condition, for the chemistry that contains these eight catalyzed reactions in addition to all the reactions of the fully connected spontaneous chemistry. At 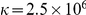 the ACS product molecules dominate over the background (the ‘background’ being defined as the set of all molecules except the ACS product molecules and the food set), in the sense that the ACS molecules have significantly larger populations than the background molecules of similar size [23]. There is a fairly sharp threshold value of 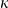 above which ACS domination appears, as evident from the comparison with the lower curve in Fig. 4A drawn for 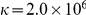. Fig. 4B shows that the steady state background concentrations decline as 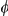 increases, while the ACS concentrations are relatively unaffected in this regime (thus ACS domination increases). If catalyzed production pathways from the food set to other molecules are broken somewhere, the concentration of the latter molecules declines significantly. This is evident from Fig. 4C for which only one reaction pair (5) is deleted from the catalyzed chemistry (which now contains no ACS) while others are catalyzed at the same strength as before.

**Figure 5:**
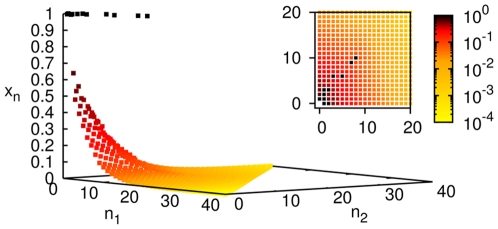
Steady state concentration profile for ACS(8,10) in a chemistry with 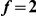. The convention is same as in Fig. 2. The molecules and reactions of the ACS are given in Table S1, the largest molecule being (8,10). 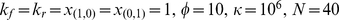.

ACS domination at a sufficiently high catalytic strength also occurs when there is more than one monomer. An example with 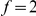 is shown in Fig. 5 whose list of catalyzed reactions is given in Table S1.

#### Understanding why ACS concentrations are large (the 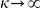 limit)

The above features are generic for a large class of ACSs. It is instructive to consider the 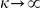 limit which we discuss analytically. When 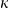 is nonzero, the terms in Eq. (1) corresponding to catalyzed reactions get modified. The net flux 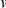 of such reaction pairs on the r.h.s. (for brevity we are omitting the subscript 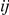 in 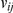) is replaced by 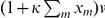, where the sum over 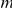 is a sum over all catalysts of the reaction pair. Now let the set 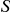 of catalyzed reactions be an ACS. Then, if 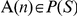 the r.h.s. of 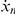 contains at least one such catalyzed term, while if 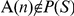 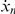 contains no such term. For example, for ACS65, we have
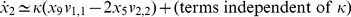
(6a)

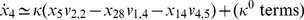
(6b)

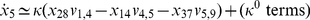
(6c)

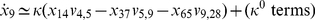
(6d)

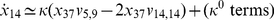
(6e)

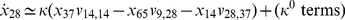
(6f)

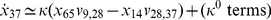
(6g)

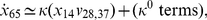
(6h)while the rate equations for all other (non ACS) molecules (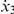, 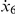, etc.) have no terms proportional to 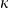. In a steady state solution the r.h.s. of Eqs. (6) is zero, and to leading order in the 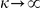 limit we must set the coefficients of 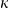 to zero. The coefficients involve only the ACS fluxes 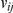 and catalyst concentrations. Each coefficient is a sum of terms, and each term is proportional to an ACS flux 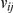. Thus 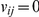 for the ACS fluxes provides a steady state solution in the 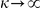 limit. Numerically we find that when 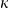 is sufficiently high the rate equations converge to this solution starting from the standard initial condition. Now 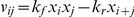, therefore 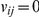 implies 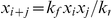 for the members of 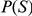. Since by definition there is a catalyzed pathway from the food set to every ACS product, we can recursively express the steady state concentration of every ACS molecule in terms of 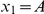: 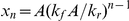.

It is evident that this argument applies whenever the set 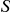 of catalyzed reactions is an ACS; thus for every member of 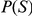, 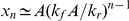 is a steady state solution of the rate equations in the limit 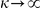. This is corroborated numerically: in Fig. 4B since 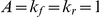, all the eight ACS products should have 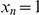 in this limit; the numerical result at 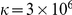 is not too far from this limiting analytical value.

#### A strong ACS counteracts dissipation

Recall from Eq. (4) and the discussion following it that every molecule in a homogeneous connected uncatalyzed chemistry has the steady state concentration 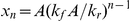 when there is no dilution flux or dissipation (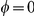), and a smaller concentration when there is dissipation (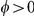). We have observed above that an ACS with a sufficiently large 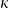 can boost the steady state concentrations of its members, even when 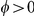, to the same level. The expression 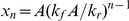 seems to represent an upper limit on the steady state concentration of 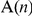, which can be approached either when dissipation goes to zero, or, when there is dissipation, by membership of an ACS whose catalytic strength becomes very large.

When the reaction pair 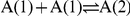 is not catalyzed the production of A(2) takes place at a much smaller rate, the spontaneous rate. Therefore its concentration is much smaller, and hence so are the concentrations of the larger molecules.

When 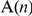 belongs to the background the r.h.s. of 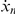 contains no term proportional to 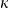, and all the 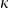-independent terms have to be kept, including the 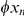 term. Thus its steady state concentration depends upon 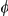, and as in the case of the uncatalyzed chemistry, declines more rapidly with 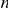 when 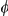 increases.

#### Multistability in the ACS dynamics and ACS domination

**Figure 6:**
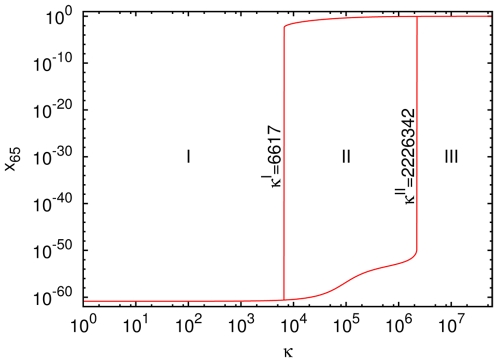
Bistability in the dynamics of ACS65. ‘Hysteresis curve’ of the steady state concentration of A(65) versus 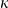 for 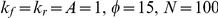. The curve is obtained by using two different initial conditions (i) the standard initial condition 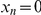 for all 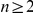, and (ii) a ‘high’ initial condition 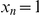 for all 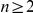. In region I (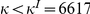) both initial conditions lead to a single fixed point in which 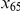 is very low, 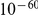. In region III (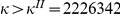) both initial conditions again lead to a single fixed point but in this fixed point 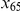 is high, close to unity. In region II (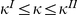) the initial condition (i) leads to the lower fixed point and (ii) leads to the upper one. The transitions are very sharp, e.g., at 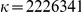 the system is numerically clearly seen in region II and at 2226343 in region III.

The reason for the sudden change in the qualitative character of the steady state profile as 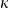 is increased is a bistability in the chemical dynamics due to the presence of the ACS. Fig. 6 shows three regions in the phase diagram of the system, separated by values 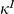 and 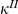 of 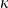. For 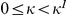 (region I), the dynamics starting from both the initial conditions mentioned in the figure caption converged to the same attractor configuration, which is a fixed point in which the large ACS molecules have a very small concentration (the concentration declines exponentially with 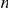). For 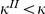 (region III), again they converge to a single attractor, a fixed point in which the ACS molecules have a significant concentration which approaches 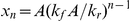 as 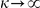. In the range 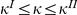 (region II), they converge to two different stable attractors, both fixed points for the ACS under discussion. (We remark that using other initial conditions we have found at least one more stable fixed point in a part of region II which has intermediate values of 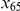, indicating that this system has multistability.)

This phase structure implies that if we start from the standard initial condition and consider the steady state profile to which the system converges for different values of 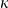, we will see a sharp change in the steady state profile as 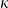 is increased from a value slightly below 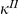 to a value slightly above 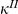. Below 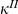 the large ACS molecules will be essentially absent in the steady state, and above 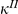 they will be present in large numbers and will dominate over the background.

Therefore, following the nomenclature of Ohtsuki and Nowak [34], who observed a similar bistability in their model with a single catalyst, we refer to 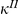 as the ‘initiation threshold’ of the ACS. Similarly 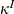 will be referred to as the ‘maintenance threshold’ of the ACS, because once the ACS has been initiated, 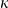 can come down to as low a value as 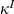, and the ACS will continue to dominate.

#### Bistability in simple ACSs

**Figure 7:**
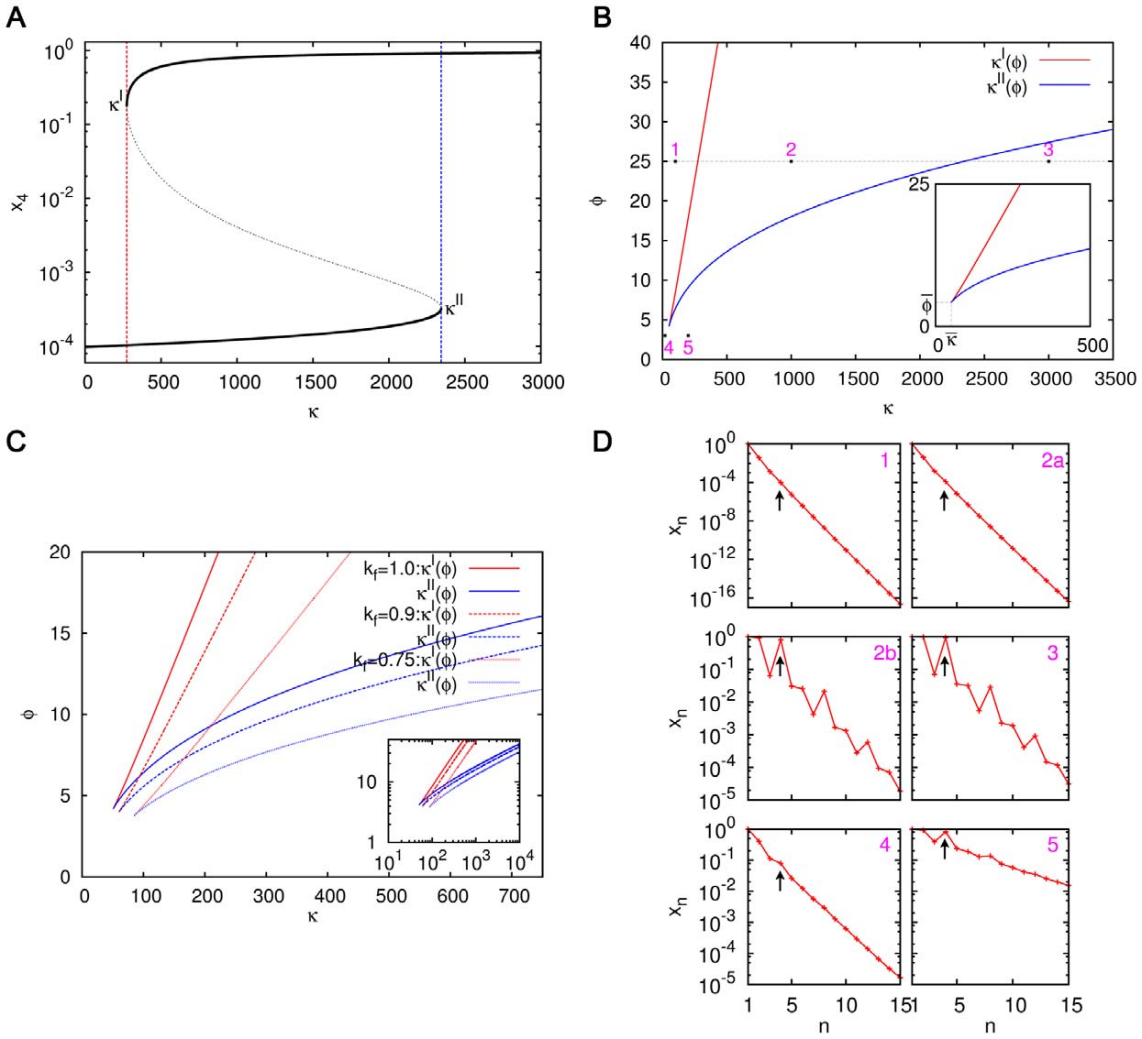
Phase diagram and concentration profiles for ACS4. (A) The steady state concentration 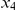 versus 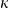 for 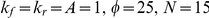. The bistable region exists for the range 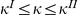 in which different initial conditions lead to two distinct steady state values of 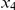. The solid black curves correspond to the two stable fixed points, and the dotted black curve to the unstable fixed point. (B) The dependence of 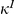 (red curve) and 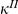 (blue curve) on 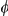 for 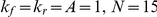. The bistable region lies between the two curves; in the rest of the phase space the system has a single fixed point. The inset shows the location of the critical point 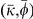; there is no bistability for 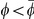. (C) Dependence of the phase boundaries on 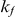 for 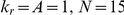, with the inset showing the behaviour on a log-log plot. (D) The steady state concentration profile of molecules shown at five representative points in the phase space (numbered 1 through 5 and marked in (B)). Note that at the phase point 2 that lies between the 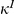 and 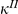 curves there are two steady state profiles corresponding to the two stable fixed points of the system. The figure marked 2a shows the profile starting from the standard initial condition, and 2b from the initial condition where 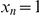 for all 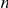. The arrows draw attention to the concentration of the catalyst, A(4).

In general 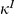 and 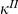 depend upon the other parameters, as well as the topology of the catalyzed and spontaneous chemistries. The phase structure is exhibited in more detail for a simpler example in Fig. 7, where the catalyzed chemistry consists of only two reaction pairs:
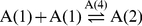
(7a)

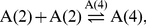
(7b)which constitute an ACS (called ACS4). This system, investigated numerically using XPPAUT, shows bistability. For a fixed 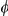 the bistability diagram is shown in Fig. 7A. The dependence of 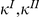 on 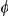 is exhibited in Fig. 7B, and on both 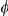 and 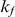 in Fig. 7C. For a given 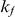, there is critical value of 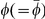 at which the 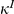 and 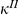 curves meet, below which there is no bistability. The locations of the phase boundaries, the 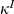 and 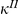 curves, depend upon the specific underlying chemistries (catalyzed and spontaneous) as well as the ACS topology. The steady state profiles are shown at sample points in the phase space in Fig. 7D. For 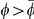 it can be seen, that as in the case of the larger ACS discussed earlier, if we start from the standard initial condition, the largest molecule of the catalyzed chemistry, here A(4), dominates over the background in the steady state only for 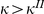 (e.g., the panel marked 3 in Fig. 7D). In the range 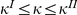, it dominates only if we start from initial conditions where it has a large enough value to begin with (panel 2b), but not if we start from the standard initial condition (panel 2a). It does not dominate for any initial condition if 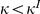 (panel 1). If 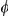 is below 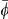, there is a single attractor with no significant ACS dominance if 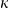 is small (panel 4), or if 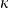 is large (panel 5), ACS dominance exists but is not very pronounced as the background concentrations are also substantial.

We remark that while bistability seems to be quite generic in homogeneous chemistries containing ACSs, the existence of an ACS does not guarantee that bistability exists somewhere in phase space. For example consider the simplest possible chemistry (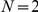) containing only the monomer (which is buffered) and the dimer. If we assume that the sole reaction pair 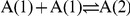 is catalyzed by 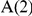, the catalyzed chemistry is trivially an ACS and the only rate equation is 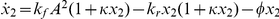. The system can be solved exactly and always goes to a global fixed point attractor starting from any initial condition 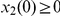. However, the 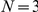 chemistry defined by the two catalyzed reactions
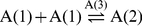
(8a)

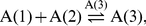
(8b)does exhibit bistability at a sufficiently large 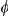. Ohtsuki and Nowak [34] had also found a lower limit on catalyst size for bistability to exist in their model. Similar results hold for the 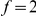 case. From our simulations a general observation seems to be that bistability is ubiquitous at sufficiently large values of 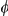 in homogeneous chemistries whenever the smallest catalyst is large enough compared to the food set. When it does exist it seems to provide a crisp criterion for ‘ACS domination’, including ‘initiation’ (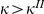) and ‘maintenance’ (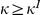).

We must mention that there exists a substantial mathematical literature on the nature of attractors in chemical reaction systems including conditions for multistability and monotonicity [46]–[50]. It would be interesting to apply some of those results to models of the kind being studied here, which involve a large number of molecular species.

### A problem for primordial ACSs to produce large molecules: The requirement of exponentially large catalytic strength

A natural initial condition for the origin of life scenario is one where only the food set molecules, and perhaps a few other not very large molecules (dimers, trimers, etc.) have nonzero concentrations, while the large molecules have zero concentrations. It is from such an initial condition that we would like to see the emergence of large molecules through the dynamics. We have seen that in uncatalyzed chemistries, the concentrations of the large molecules remain exponentially small (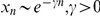). In catalyzed chemistries, especially in the presence of an ACS, a few specific large molecules produced by the ACS can acquire a high population. However, this seems to require a large catalytic strength for the catalysts. For example, for ACS65 this happens at 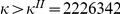, starting from the standard initial condition. The fact that such a large catalytic strength is needed to produce appreciable concentrations of molecules of even moderate length like 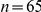 could be a problem for the ACS mechanism to produce large molecules in the kind of prebiotic scenario we are considering. In this section we characterize the problem somewhat more quantitatively by determining how 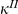 depends upon the size 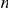 of the catalysts in the ACS.

As mentioned earlier, the values of 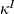, 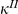 depend on the topology of the ACS. The topology of the ACS includes the set of catalyzed reactions and the assignment of catalysts to each of the catalyzed reactions. Define the ‘length’ 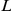 of an ACS as the size of (i.e., the total number of monomers of all types in) the largest molecule produced in the ACS. An ‘extremal’ ACS of length 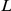 will be referred to as one in which all reactions belonging to the ACS are catalyzed by the same molecule which is the largest molecule (of size 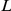) in the ACS. For concreteness, since we are interested in the dependence of 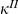 on the catalyst size, we consider only extremal ACSs of length 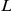. We assume that the catalyst has the same catalytic strength 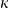 for all the reactions in the ACS. We wish to determine the bistable region for such ACSs and in particular how the values of 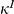 and 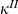 depend upon 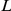. These values depend upon the precise set of catalyzed reactions constituting the ACS. For illustrative purposes we consider three different ways of generating the ACS described under Methods as Algorithm 1, 2 and 3, which generate ACSs with different characteristic structure.

**Figure 8:**
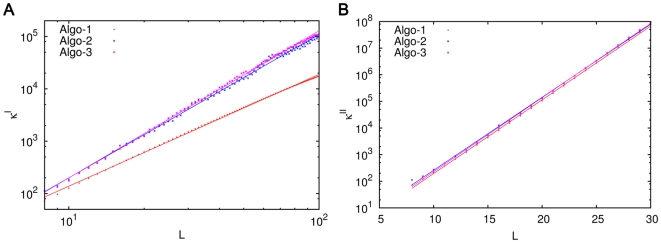
The dependence of the bistable region on catalyst length 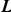. (A) The dependence of 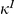 on 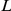. (B) The dependence of 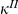 on 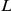. Simulations were done for extremal ACSs of length 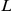 generated by three algorithms (see Methods), represented in the figure by different colours. For each 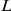 the ACS in question has the property that the largest molecule produced in the ACS has 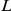 monomers and catalyzes all the reactions in the ACS. All simulations were done for 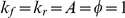. 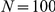 in all cases except the 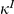 curve for Algorithm 1, where 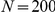, because in this case ‘finite-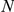
’ effects were quite significant at 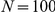. The figures suggest an approximate power law growth of 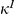 and exponential growth of 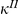 with 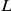.

We determine the 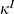 and 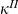 values for ACSs of different values of 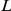 numerically. These are plotted in Fig. 8. It is evident that 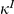 increases with 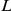 according to a power law 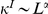 (with 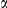 ranging from 2.1 to 2.8 for the three algorithms), while 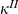 increases exponentially,
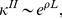
(9)with 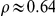 for all the algorithms. 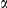 and 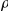 depend upon the other parameters. In particular we find that 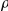 increases with 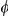, i.e., the catalytic strength needed for large molecules to arise increases faster with the size of catalyst at larger values of dissipation. This generalizes, to a much larger class of models, the results of Ohtsuki and Nowak [34], who found a linear dependence of 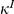 on 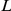 and an exponential dependence of 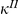.

The exponential increase of the initiation threshold, 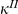, with 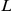, quantifies the difficulty in using ACSs to generate large molecules in the primordial scenario of the type modeled above. This means that one needs large molecules with unreasonably high catalytic strengths to exist in the chemistry in order to get them to appear with appreciable concentrations starting from physically reasonable primordial initial conditions.

### Nested ACSs: Using a small ACS to reinforce a larger one

We now discuss a mechanism that may overcome the barrier of large catalytic strengths, and may enable large molecules to arise from primordial initial conditions without exponentially increasing catalytic strengths. This mechanism relies on the existence of multiple ACSs of different sizes in the catalyzed chemistry, in a topology such that the smaller ACSs reinforce the larger ones, thereby enabling large molecules to appear with significant concentrations without exponentially increasing their catalytic strength.

**Figure 9:**
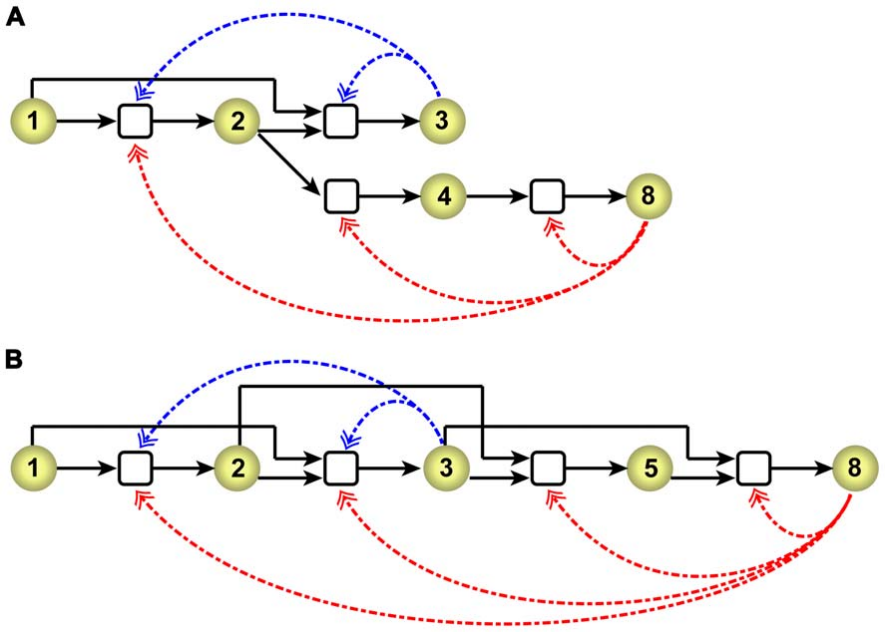
Pictorial representation of nested ACSs. (A) ACS3+8, defined by Eqs. (10) and (11). (B) ACS3+8′, defined by Eqs. (10) and (12). The notation is the same as for Fig. 3. The dashed arrows (catalytic links) are given in two colours, blue and red, to distinguish the two ACSs whose catalysts are molecules A(3) and A(8), respectively. Reactions having two catalysts are given by two distinct equations in the text (e.g. (10a) and (11a)), but in the figure are represented by a single reaction node with two incoming catalytic links (the reaction node is not duplicated to avoid visual clutter).

**Figure 10:**
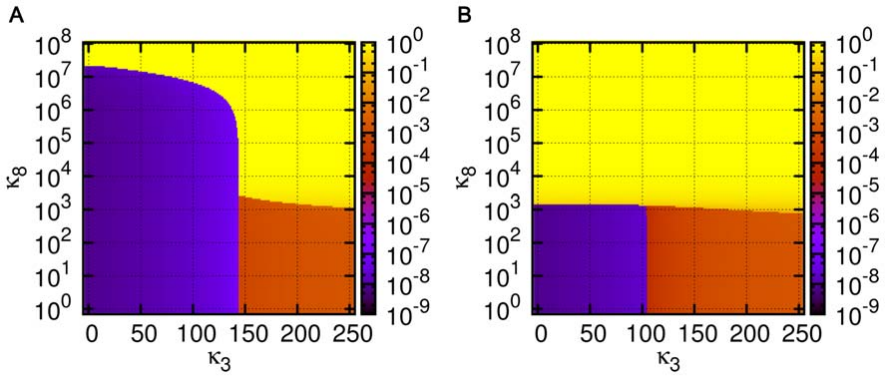
Reinforcement of a larger ACS by a smaller one: The case of ACS3+8. The figure shows the steady state concentration 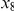 (in colour coding as indicated) for two different initial conditions as a function of 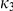 and 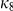, the catalytic strengths of 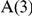 and 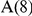 respectively. All simulations were done for 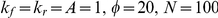. The two figures (A) and (B) differ in the initial condition of the dynamics. (A) The standard initial condition, (B) initial condition 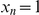 for all 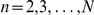.

To illustrate the basic idea we consider the following simple example where the catalyzed chemistry contains only two ACSs, one of length three and the other of length eight (which we refer to as ACS3 and ACS8, respectively), each generated by the Algorithm 2 mentioned above. All reactions of the former are catalyzed by 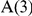 with a catalytic strength 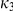, and of the latter by 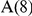 with the catalytic strength 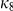. Thus the two ACSs are:
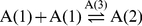
(10a)

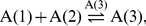
(10b)and
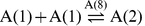
(11a)

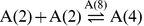
(11b)

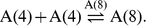
(11c)The catalyzed chemistry consists of the above five catalyzed reaction pairs (we will refer to this catalyzed chemistry as ACS3+8). This is pictorially depicted in Fig. 9A. The system also exhibits bistability, and the concentration of 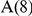 in the two fixed point attractors is exhibited in Fig. 10 as a function of 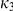 and 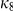.

When 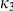 is small the two pictures in Fig. 10 show the usual bistability of ACS8 along the 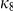 axis. The initiation and maintenance thresholds are 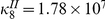 and 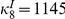 given by the location of the boundary between the low concentration region (blue, 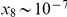) and the high concentration region (yellow 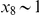) along the 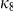 axis in Figs. 10A and 10B respectively. As 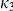 increases, the initiation threshold of ACS8 decreases slowly for a while, then drops sharply near 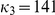. This value of 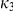 is the initiation threshold of ACS3 when 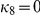. When 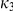 exceeds this value, the steady state value of 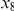 is either high (yellow, 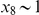) or intermediate (orange, 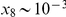), depending upon the value of 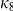.

The key point is that the initiation threshold of the larger catalyst depends on the catalytic strength of the smaller catalyst. The former plummets sharply when the latter approaches the initiation threshold of the smaller catalyst, dropping to a much lower value than before (compare the lower limit of the yellow region in Fig. 10A to the left and right of 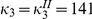; the value of 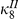 plunges several orders of magnitude from 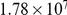 at 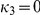 down to 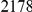 at 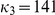). Starting from the standard initial condition, thus, the larger catalyst can acquire a significant concentration at a much lower value of its catalytic strength in the presence of a smaller ACS operating above its initiation threshold than in its absence.

### Why a small ACS reinforces a larger one

We now present an intuitive explanation of the above mentioned property. The argument rests on two observations.

#### (a) Why the initiation threshold is exponentially large

**Figure 11:**
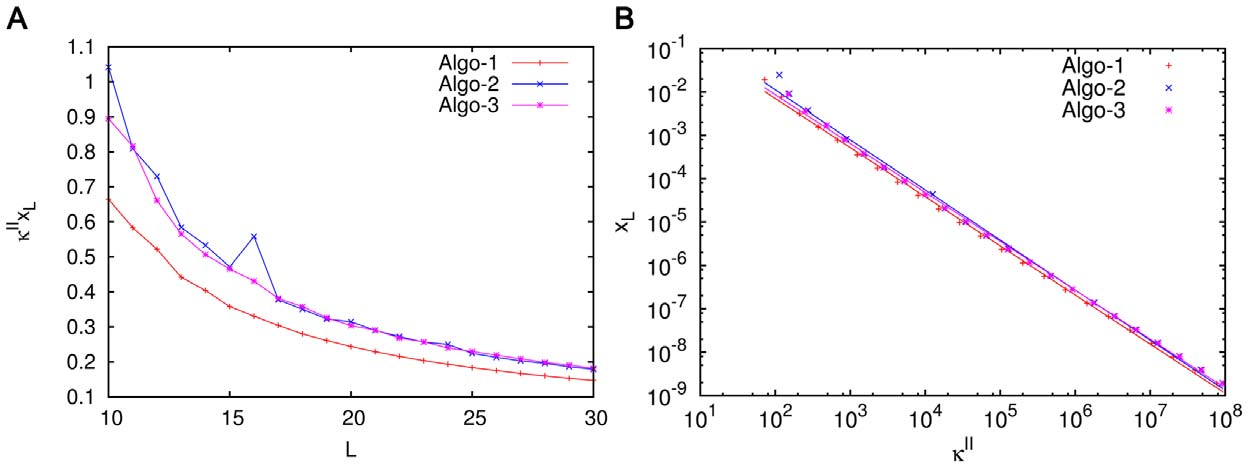
The product 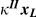 is of order unity. This figure is produced from the same data as was used for Fig. 8. Simulations were done for chemistries containing extremal ACSs of length 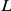 generated by the three algorithms discussed earlier, represented in the figure by different colours. For this figure each chemistry was simulated at a value of 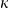 equal to the initiation threshold 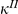 corresponding to that chemistry, and the steady state concentration 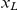 of the catalyst was determined in the low fixed point (starting from the standard initial condition). The parameters values are the same as in Fig. 8. (A) The product of 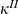 and 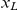 as a function of 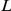. (B) 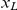 versus 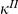 on a log-log plot. The slopes of the fitted straight lines vary in the range −1.13 to −1.16 for the three algorithms (slope = −1 would have meant that 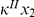 is strictly constant. The figure shows that while each individual factor 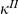 and 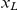 ranges over several orders of magnitude, their product, though not constant, is of order unity.

The first observation attempts to explain why 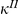 is so large in the first place. The contribution of a catalyst to the rate of the reaction it catalyzes appears through the factor 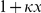, where 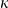 is the catalytic strength of the catalyst and 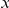 its concentration. The term unity in the above factor is the relative contribution of the spontaneous (uncatalyzed) reaction rate. If the catalyst is to play a significant role in the reaction, the catalytic contribution to the reaction rate should be at least comparable to the spontaneous rate, i.e., 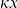 should be at least comparable to unity. As we have seen earlier the concentration of large molecules is typically damped exponentially with their size. Therefore the compensating factor 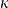 needs to increase exponentially in order for the catalyzed reaction rate to be comparable to the spontaneous reaction rate. For concreteness consider the extremal ACSs of length 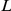 and consider the steady state population 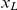 of the catalyst 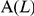 in the low fixed point as 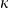 is increased. In the spirit of this rough argument one expects that at the initiation threshold the term 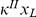 should be of order unity. In Fig. 11 we display this product for different values of 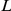. Though there is a secular decreasing trend with 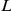, this product remains of order unity (Fig. 11A) even as the individual factors change over several orders of magnitude (Fig. 11B). This lends numerical support to the above explanation for the exponential dependence of 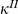 on 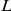.

#### (b) Role of the background and spontaneous reactions

The second observation is that when 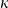 exceeds the initiation threshold for a catalyzed chemistry containing an ACS, not only do the steady state concentrations of the ACS product molecules rise by several orders of magnitude, but also those of the background molecules rise. As an example compare the two steady state profiles of ACS65 in Fig. 4A, which correspond to values of 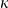 below and above the initiation threshold. As one goes from the lower to the upper curve, the concentration of the ACS members of course increases dramatically (as shown by the sharp peaks), but note that the concentrations of other molecules not produced by catalyzed reactions also goes up significantly. Thus in the chemistry containing two ACSs (ACS3+8) as one moves along the 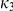 axis in Fig. 10A and crosses the initiation threshold of ACS3 (i.e., 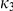 exceeds 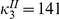), the concentration of 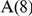 (a molecule belonging to the background of ACS3 as its production is not catalysed by ACS3) increases from 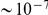 (blue region) to 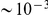 (orange region). This increase in the concentration of 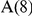 by a factor of 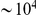 makes it easier for ACS8 to function and its initiation threshold drops by a corresponding factor of about 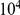 (from 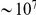 to 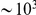).

This fact highlights the role of spontaneous reactions in the overall dynamics. The background molecules are connected to the ACS through spontaneous reactions, and if it were not for the latter, an ACS would not be able to push up the concentrations of its nearby background. We shall refer to a structure such as the one described above containing ACSs of different sizes with the smaller ACS feeding into the larger one through the spontaneous reactions as a ‘nested ACS’ structure.

### The role of ‘overlapping’ catalyzed pathways in nested ACSs

The above example also serves to highlight some other features of catalyzed chemistries containing multiple ACSs. Note that the production pathway of 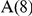 in ACS8 (Eqs. 11 and Fig. 9A) contains one reaction pair in common with ACS3, namely the reaction pair 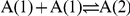. One can consider a situation wherein the overlap is greater. E.g., consider the ACS8′ defined by
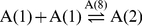
(12a)

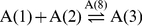
(12b)

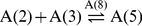
(12c)

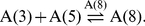
(12d)Now the set of reactions in ACS3 is a subset of ACS8′ (ignoring the catalyst, which is different in the two cases). The degree of overlap of the catalyzed reaction sets between a pair of nested ACSs makes a difference in the dynamics. Consider, for example, the catalyzed chemistry consisting of ACS3 and ACS8′, i.e., the set of catalyzed reactions given by Eqs. (0) and (0), which we refer to as ACS3+8′. This is picturized in Fig. 9B. Like ACS3+8, this chemistry also shows a reduction of 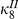, when 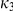 exceeds its initiation threshold. We find that while at 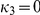 the value of 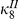 for the two chemistries is not too different (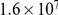 for ACS3+8′ versus 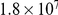 for ACS3+8), at 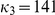, 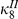 reduces to a value 920 in ACS3+8′, which is less than half of the value 2178 that it reduces to in ACS3+8. Thus a larger degree of overlap between the catalyzed reaction sets of nested ACSs causes more effective reinforcement.

**Figure 12:**
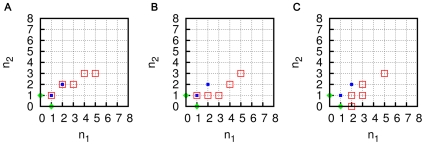
Examples of nested ACS pairs with different degrees of overlap for 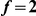. In the three cases the reaction sets have (A) maximal overlap, (B) partial overlap, (C) no overlap. The blue and red squares marking the grid points indicate the identity of molecules produced in the two ACSs; blue filled squares correspond to the products of the smaller ACS, red unfilled squares to those of the larger ACS. The 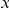 and 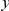 axes denote the number of monomers of type (1,0) and (0,1), respectively, in the molecules. The green rhombuses represent the two monomers.

Another example with this behaviour for 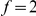 is described in Fig. 12 (for a pictorial representation of the network see Supporting Fig. S1). In each of the three ACS pairs shown in the figure, the smaller ACS, of length 4, is the same, (it will be referred to as ACS(2,2)) and is defined by the reactions (each catalyzed by (2,2))
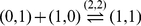
(13a)

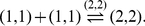
(13b)The three larger ACSs, called ACS(5,3)(a), ACS(5,3)(b) and ACS(5,3)(c), respectively, can essentially be determined from the figure. For example, ACS(5,3)(a) consists of the two reaction pairs given by Eqs. (13), both catalyzed by (5,3) as well as the three reactions
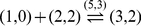
(14a)

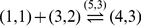
(14b)

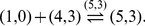
(14c)ACS(5,3)(b) consists of the single reaction pair given by the first of Eqs. (13), catalyzed by (5,3), as well as the four reactions
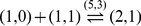
(15a)

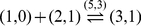
(15b)

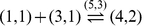
(15c)

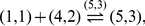
(15d)and ACS(5,3)(c) consists of the five reaction pairs
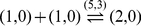
(16a)

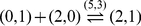
(16b)

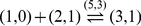
(16c)

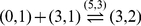
(16d)

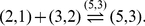
(16e)


We consider the population dynamics of chemistries in which the spontaneous part includes all possible ligation and cleavage reactions involving molecules with upto 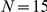 monomers with homogeneous rate constants 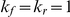, 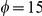, and the catalyzed part containing one or more of the above mentioned ACSs. When ACS(2,2) is the only ACS present, the system shows bistability with the initiation threshold being 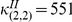. When ACS(5,3)(a), (b) or (c) are the only ACSs present, the initiation thresholds for them are 1125197, 1031082, and 1000112, respectively. When ACS(2,2) and one of ACS(5,3) (a), (b) or (c) are both present, and the catalytic strength of (2,2) is 552, the initiation thresholds of the three larger ACSs reduce to 941, 1256, and 2482, respectively. Again, it is seen that the larger the degree of overlap of the two nested ACSs, the more effective is the reinforcement.

### A hierarchy of nested ACSs: A possible route for the appearance of large molecules

The process of nesting discussed above for two ACSs can be extended to multiple levels of ACSs connected to each other. Here we discuss sequences of ACSs of increasing size, with the catalyzed reaction set of each ACS in the sequence partially or completely contained within the next one, and the catalytic strength of molecules increasing with size in a controlled manner. We construct examples of such sequences in which large catalyst molecules containing several hundred monomers can acquire significant concentrations starting from the standard initial condition, even though all catalysts have moderate catalytic strengths.

In order to construct a cascade of nested ACSs in which reaction sets of smaller ACSs are completely contained in the larger ones (maximal overlap), we used Algorithm 4 described in Methods. This algorithm produces a cascade of ACSs with 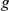 steps (generations), with the 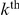 generation ACS containing 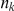 new reactions. We studied several catalyzed chemistries containing a cascade of nested ACSs for 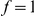 and 2. One example of each type is presented below; other examples gave qualitatively similar results.

### Dominance of an ACS of length 441 (ACS441)

**Figure 13:**
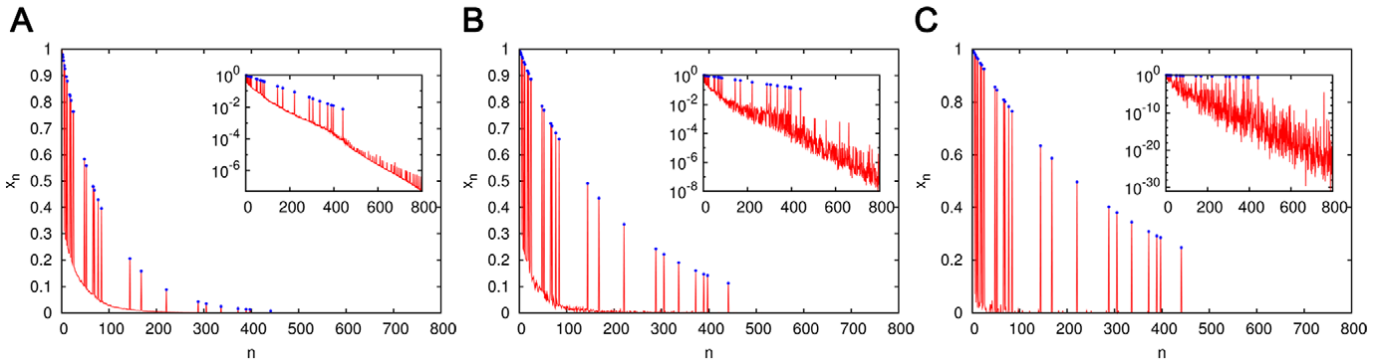
The dominance of a cascade of nested ACSs with a molecule of size 441 (ACS441). The molecules and reactions of this ACS are listed in Table S2. The red curves show the steady state concentration 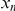 of all the molecules as a function of their size 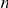, starting from the standard initial condition; blue dots show the concentrations of the ACS molecules. Insets show the same on a semi-log plot. It is evident that the large ACS molecules acquire a significant concentration. The catalytic strengths of the ACS molecules depend upon their size 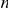 according to 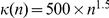, and for all cases 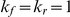, 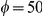, 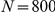. The three figures differ in the level of sparseness of the spontaneous chemistry in which the ACS is embedded. The spontaneous chemistry in (A) is fully connected, in (B) has degree 20, and in (C) has degree 2.

For 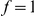 we describe a cascade with 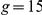 and 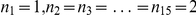. This catalyzed chemistry had 29 product molecules, the largest of which was 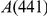 having 441 monomers. The list of molecules and reactions is given in Table S2. The catalytic strength 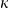 of each molecule was chosen by an explicit length dependent rule
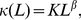
(17)where 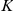 and 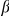 are constants. We describe a simulation with 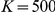 and 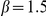. This particular rule was chosen to contrast with Eq. (9) which characterizes the initiation threshold of an extremal ACS of length 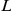. For a value of 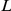 such as 441, the exponential function in Eq. (9) would have given an astronomically large catalytic strength, whereas the much slower growing power law in Eq. (17) gives 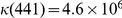 for the above mentioned values of the constants. Starting from the standard initial condition, the steady state concentration profile of this catalyzed chemistry embedded in a fully connected spontaneous chemistry with 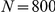 is shown in Fig. 13A. This example shows that with the nested ACS structure in the catalyzed chemistry, large catalyst molecules can acquire significant concentrations starting from an initial condition containing only the monomers, even when catalytic strengths grow quite slowly with the length of the catalyst. It is worth noting that product of the catalytic strength of 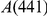 and its steady state concentration (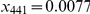) is about 36000, and this is the factor by which it speeds up the reactions it catalyzes over the spontaneous rate. In view of the fact that enzymes containing a few hundred amino acids speed up the reactions they catalyze within cells by factors of about 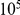 and greater, the catalytic efficiency demanded of 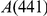 does not seem unreasonably high.

Eq. (17), which gives a particular functional form for 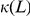, is ad-hoc, and, at this stage, merely an example given to quantify the level of catalytic strengths that is sufficient for large molecules to arise in appreciable concentrations in the prebiotic scenario under consideration if chemistry has the nested ACS structure of the kind discussed. One may ask if an even weaker requirement would suffice. We have considered smaller values of 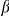 (1.2 and 1.0) keeping 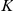 fixed, and found that ACS molecules upto a particular size (depending on 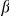) do well but that the concentration of larger ACS molecules trails off and merges with the background. The size range of ACS molecules that do well can be increased by increasing the coefficient 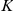. Since the results depend upon several factors, including the topology of the ACS and the uncatalyzed chemistry, a detailed investigation has not been carried out.

### Effect of a ‘sparse’ spontaneous chemistry

Fig. 13 also shows another aspect of ACS dynamics – the relationship between ACS domination and the sparseness of the uncatalyzed chemistry. A fully connected uncatalyzed chemistry is one in which all possible ligation and cleavage reactions are allowed. A chemistry with average degree 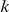 is one in which the average number of ligation reactions in which a molecule can be produced is 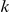. In a fully connected 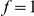 chemistry, a molecule of size 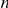 can be produced in about 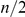 ligation reactions (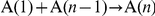, 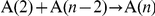, etc.); therefore the average degree of a chemistry containing all molecules upto size 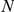 is about 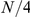 ( = 200 for the chemistry in Fig. 13A). In Figs. 13B and 13C, we pruned the uncatalyzed reaction set to only 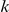 ligation reactions per molecule (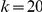 and 2, respectively), randomly chosen from all possible ligation reactions producing the molecule. (For molecules too small to have 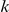 ligation reactions, all ligation reactions were retained. For the ACS molecules the ligation reaction producing them in the catalyzed chemistry was included as one of the 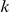 uncatalyzed reactions.) Note that while we refer to only the ligation reactions and not cleavage reactions for the purpose of defining the degree of a molecule, in our simulations all reactions are treated as reverse reactions. That is, for every ligation reaction included in the chemistry the reverse (cleavage) reaction is also present. It is seen in Fig. 13 that the increase of sparseness causes the background concentrations to decline. This is because there are fewer pathways to produce the background molecules. There is also a larger variation in their concentrations because their production reactions are chosen randomly, and background molecules produced in reactions that happen to involve the ACS molecules as reactants do better than others. The ACS molecules are seen to dominate more strongly over the background in sparser chemistries; this is because there are fewer production pathways to the background that drain their concentrations.

### Cascading nested ACSs with 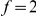


**Figure 14:**
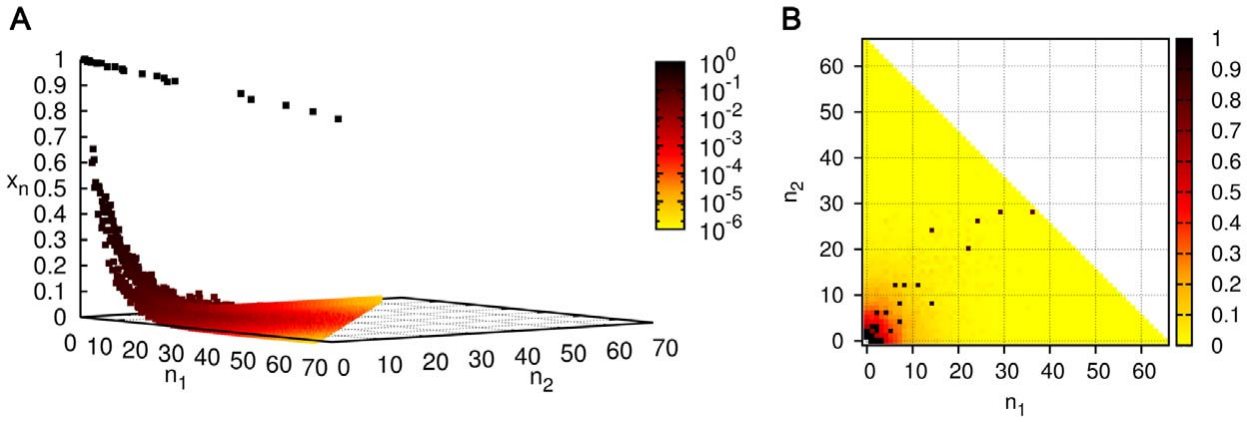
Dominance of a cascade of nested ACSs with length 64 (ACS(36,28)) in a 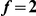 chemistry. (A) 3D plot showing the steady state concentration 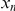 of the molecule 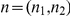 as a function of 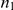 and 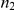, starting from the standard initial condition. The colour coding is on a logarithmic scale of the concentration. (B) A ‘top view’ of the same so that the ACS molecules and background are more clearly distinguished. The colour coding here is on a linear scale of concentration. The food set and ACS molecules have the highest concentrations and stand out as black dots. The catalytic strengths of the ACS molecules depend upon their size 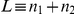 according to 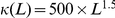, and 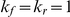, 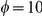, 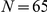. The spontaneous chemistry has degree 20.

An example of a nested ACS with two food sources, ACS(36,28), is presented in Fig. 14. This is also generated by Algorithm 4 and has 7 generations with 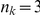 for each generation, the largest molecule being (36,28) (the full list of molecules and reactions is given is Table S3). Again starting from the standard initial condition the larger ACS molecules acquire appreciable concentrations with a moderate demand on their catalytic strengths.

**Figure 15:**
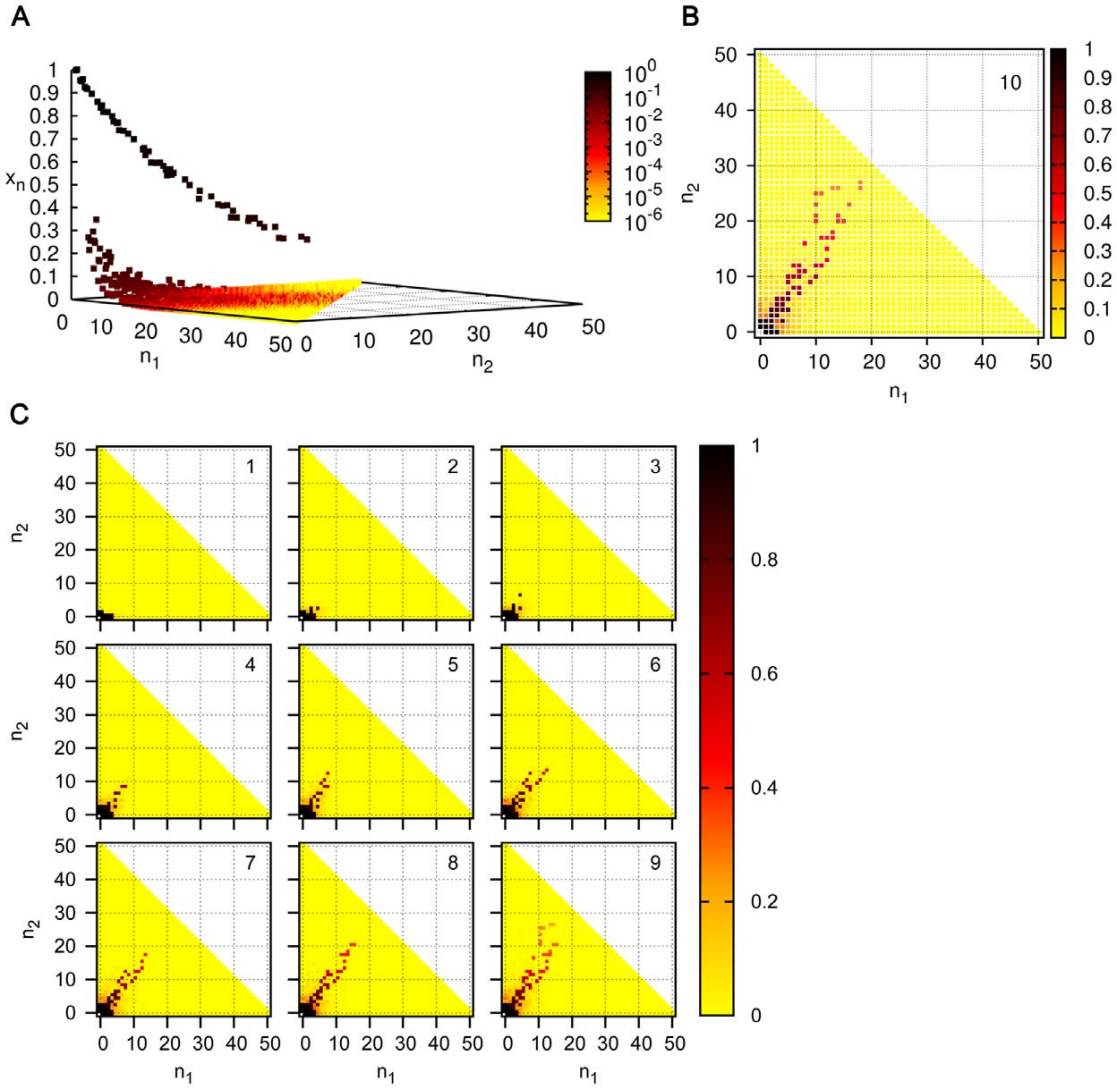
Dominance of a cascade of partially overlapping nested ACSs (ACS(18,27)). (A) Steady state concentration profile starting from the standard initial condition. (B) Top view of the same. (C) Sequence of steady state concentration profiles as each successive ACS is added to the chemistry. The legend for (A) is the same as for Fig. 14A and for (B) and (C) the same as Fig. 14B. 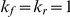, 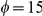, 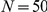, and the spontaneous chemistry has degree 5.

As a final example we present in Fig. 15 a cascade of ACSs, named ACS(18,27) after its largest molecule, in which smaller ACSs have only a partial overlap with longer ones. This is generated using Algorithm 5 (see Methods) and consists of a series of 10 ACSs of increasing length. The detailed list of molecules, reactions and catalytic strengths is given in Table S4. Each successive ACS in the cascade has only a few reactions in common with other ACSs. Unlike in the examples discussed above, generated by Algorithm 4, in the present case molecules (except the small molecules) produced in the catalyzed chemistry have typically only one or two catalyzed ligation reactions producing them. The chemistry also contains a number of catalyzed ‘side reactions’, which produce molecules that are neither catalysts nor reactants in any pathway leading to the largest molecule. In fact there is a ‘side pathway’ consisting of several reactions that may be viewed as ‘draining the resources’ of the main ACS. ACS dominance at moderate catalytic strengths occurs for this chemistry also. The largest 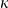 is 50000 for the molecule (18,27), and at a steady state population of 0.26 enhances the rate of a reaction by a factor of 13000 over the spontaneous rate. This shows that the mechanism outlined by us is not restricted to maximally overlapping nested ACSs but is more generic.

## Discussion

Our work discusses a possible mechanism by which large molecules can arise in a prebiotic scenario. In the context of the present model the appearance of large molecules is a natural dynamical consequence of chemistry possessing the structure described above – a cascading nested ACS structure (with a not very demanding set of catalytic strengths) embedded in a spontaneous chemistry – together with the buffered presence of the food set molecules in a well stirred region of space. The mechanism is an incremental one: at each step successive step the system is able to access new states made available by the previous step while making only an incremental demand on molecular catalytic capabilities.

The kind of mathematical model we have studied, inspired by the work of Bagley and Farmer, is quite abstract; its virtue is the economy of assumptions that go into its structure. The main ingredients are that objects can combine with each other in processes or ‘reactions’ to form other objects and certain objects can facilitate certain processes, i.e., ‘catalyze’ certain reactions. The population dynamics implements a simple scheme for how the abundances of the objects would change with time assuming that the probability of objects combining is proportional to their abundances. Such a generic scheme while it applies in detail to no particular situation allows us to imagine mechanisms at a conceptual level. It is significant that in this scheme an ACS can direct the flows towards itself and cause a certain sparse subset of objects, including some specific large composite ones, to capture a large fraction of the chemical resources.

At this level of abstraction the model (or a variant with qualitatively similar features) could apply to the peptide chemistry as well as an RNA chemistry and to a prebiotic metabolism, as already noted by Bagley and Farmer. Indeed it would be equally applicable if a prebiotic environment actually had a mixture of ingredients from all these classes of chemistries, a possibility that has been advocated in, e.g., [51], [52]. Copley, Smith and Morowitz [51] have proposed a scenario which seeks to explain how the RNA world might have originated through a series of incremental steps starting from a primitive metabolism. The food sources for this supposed metabolism are 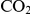, 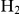, 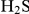, 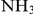, etc., in a hydrothermal vent. Their scenario envisages multiple stages of increasing complexity which they refer to as (i) the monomer stage, in which metabolism, possibly powered by an autocatalytic set such as the reverse TCA cycle, produces nucleotides and simple amino acids, (ii) the multimer stage, which produces dimers and small cofactors, (iii) the micro-RNA stage, producing of oligonucleotides of length 3–10, (iv) the mini-RNA stage, with 11–40 mers, followed by (v) the macro-RNA stage, or the RNA world. In their scenario each successive stage produced better catalysts that collectively catalyzed not only their own production from the molecules of the previous stage, but also the reactions of the lower stages. This structure is very similar to the cascade of nested ACSs that we have discussed. A suitably modified version of our model could be constructed to explore the dynamics of this scenario in more detail. At a general level, in the fact that the dynamics of our model results in the stable domination, or concentrating, of the large catalysts, our mathematical work perhaps lends support to the workability of such a scenario.

There is another level at which the present model (or its variants) might talk to prebiotic chemistry. Morowitz [53] has suggested that the metabolic network itself has a shell like structure which can convert simple molecules like 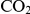, 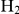, 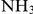, etc., through “a hierarchy of nested reaction networks involving increasing complexity” into purines, pyrimidines, complex cofactors, etc. Reaction sets created by our Algorithms 4 and 5 are reminiscent of this picture. Missing from Morowitz's picture is a catalyst assignment for each reaction from among the molecules in the various shells or from among other catalysts accessible prebiotically (e.g., surfaces in hydrothermal vents). It might be worthwhile to attempt to add in that information for a more complete scenario and for potential modeling.

### Caveats and future directions

(1) We have studied the properties of autocatalytic systems, by choosing specific examples of ACS topology and special algorithms for constructing them. This has allowed us to systematically investigate the dynamical properties that ACSs offer. We believe that similar dynamical properties would hold for more general topological structures than we have considered. Nevertheless the question arises as to whether all these structures are very special structures and whether or not they are likely to arise within real chemistry and ‘generic’ artificial chemistries. ACSs have been shown to be quite generic in a large class of randomly constructed artificial chemistries [54], [55], and a similar analysis could be extended to cascading nested ACSs. This would help parametrize or characterize chemistries that would contain such structures and those that would not. In this context it would useful to go beyond the simple case we have considered in which a molecule is defined by the number of monomers of each type and not their sequence. It may also be interesting to look for structures similar to nested ACSs in real metabolic networks using methods similar to those in [56].

(2) An important related question is one of side reactions (discussed in [57], [58]) which might destroy the efficacy of ACSs. In the real chemistry one expects that even if cascading nested ACSs exist, there would also exist other catalyzed reactions channelizing the ACS products into pathways leading in other directions. Whether substantial populations of large molecules in the nested ACSs can be maintained in the presence of such diversion is a question that remains to be systematically investigated. Our last example of cascading nested ACSs in fact has several side reactions and it may be noted that large ACSs still dominated in that case. We remark that while side reactions can drain resources from ACSs, they also help the system to explore new directions in chemical space in an evolutionary scenario.

(3) We have considered deterministic dynamics in this paper. Stochastic fluctuations are important when molecular populations are small. For a chemistry containing multiple ACSs, Bagley, Farmer and Fontana [24] used stochasticity to produce examples of trajectories that differed from each other in the sequence of ACSs that came to dominate the reactor. It would be interesting to explore such effects in the context of the present model.

(4) Another simplification we have made is that of homogeneous chemistries, wherein the rate constants of all spontaneous reactions have been taken to be the same, and even catalytic strengths, where variable, have been taken to be smooth functions of the length. We have checked that introducing a small amount of heterogeneity or randomness in the rate constants does not change the qualitative behaviour significantly. However, the effect of cranking up the heterogeneity has not been studied. From studies of disordered systems in statistical mechanics and condensed matter physics it has become clear that such heterogeneity can lead to rugged landscapes, multiple attractors and timescales, and paths that are difficult to locate [59]. The dynamics of such systems when they are driven by a non-equilibrium flux or buffering of food set molecules is an open question. It is possible that the constraints placed by the ruggedness of the landscape will reduce the number of accessible paths. The populating of molecules at different levels in a nested hierarchy of ACSs is likely to happen in fits and starts on multiple timescales when heterogeneity is accounted for. It is perhaps in such a scenario that one should look for answers to the questions raised under (1), (2) and (3) above.

## Methods

### A. Generating extremal ACSs of length 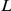


In the following we describe three different algorithms used for generating a set of reactions that provide a pathway to produce a molecule of a given length 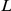 from the food set.

#### Algorithm 1: Incremental, smallest-stepsize, deterministic construction

Each molecule of size 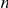 (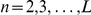) is produced in the reaction 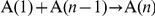. All such reactions for 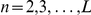 are included in the ACS.

#### Algorithm 2: Shortest path, top-down, deterministic construction

Start with 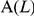. If 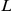 is even, it is produced in the reaction 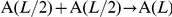. If 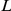 is odd, say 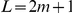, then 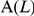 is produced in the reaction 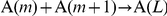. The same algorithm is used to select a reaction for the production of the precursor(s) of 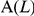 (namely, for 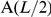 if 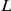 is even, and for each of 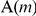 and 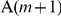 if 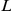 is odd (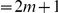)), and is iterated for their precursors, etc., until the only reactant appearing in the reactions is the food set molecule, A(1). All the production reactions mentioned above are included in the ACS.

#### Algorithm 3: Incremental, random construction

In this method, starting from the food set 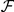 the set of reactants 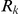 is sequentially enlarged step by step (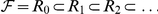) to include larger molecules and a reaction chosen randomly until a product of length 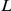 is obtained. 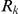 denotes the set of reactants at step 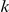 in the algorithm, and 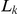 the size of the largest molecule in 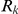. At step 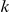, pick a pair of molecules (say 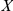 and 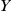) randomly from 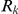, and determine the product 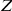 formed if they were to be ligated (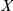 being the same as 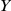 is allowed). If the size of this product, 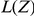, is 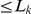 or 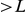, discard the pair and choose another pair. If 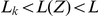, add the ligation reaction 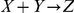 to the ACS, define 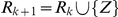, and iterate the procedure. If 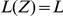, add the ligation reaction 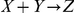 to the ACS, and stop. Initially (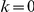) the reactant set is just the food set, 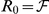.

To complete the extremal ACS, we assign 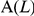 as the catalyst of all the reactions generated using any of the above algorithms. For the simulations reported in the paper, the reverse of each reaction is also included as a reaction catalyzed by the same catalyst. For concreteness we have described the Algorithms 1 and 2 for the single monomer case; their generalizations to 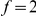 have also been considered by us. Algorithm 3, as described above, can be used for any 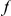.

### B. Generating a cascade of nested ACSs

#### Algorithm 4: Incremental, random construction of a sequence of reaction sets with maximal overlap

We construct successive sets, or ‘generations’, 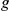 in number, 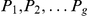, of product molecules, starting from the food set 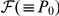. Each generation 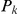 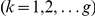 has a pre-specifed number of molecules, 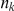 (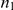, 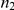, 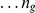 need to be specified before running the algorithm). At step 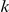 of the cascade an ACS 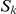 is constructed from the previous ACS 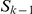 by adding reactions between molecules belonging to a reactant set 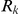 consisting of all the products of the previous generations and the food set, 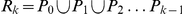. Let 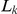 denote the size of the largest molecule in 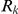. At the beginning of the 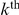 step, 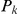 is empty and the set of reactions in 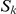 is the same as in 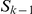, except that the catalysts of the reactions in 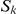 are not yet assigned. (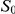 is the empty set.) To construct 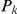 and 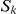, pick a molecule 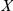 at random from the previous generation of products 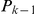 and another molecule 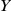 at random from 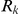, and determine the product 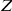 formed if they were to be ligated (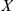 being the same as 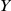 is allowed). If the size of this product, 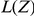, is 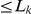, discard the pair and choose another pair. If 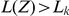, add the molecule 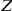 to 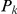 and the ligation reaction 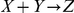 to 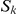. Repeat this procedure until 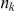 molecules are added to 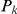 and 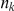 reactions are added to 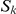. Assign catalysts to each reaction in 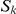 (which includes the reactions inherited from 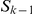 and the new 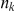 reactions) randomly from 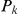. This completes the 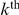 step. To get the full cascade with 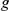 generations this process is carried out for 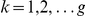. By construction the set of reactions (ignoring the catalyst) of each 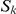 is fully contained in that of 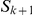 but the catalysts are different, being drawn from 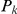 for 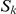 and 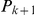 for 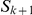. The union of the 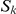
's constitutes the set of reactions in the catalyzed chemistry. Note that in this catalyzed chemistry reactions have multiple catalysts, the multiplicity declining for reactions producing higher generation molecules.

The size of the food set constrains how large 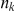 can be. For 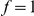, 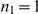 and 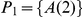 as the only product one can make from a reaction in the food set is 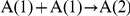, and 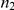 can only have values 1 and 2 as the only products one can produce in the second generation (from reactants in 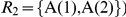 are A(3) and A(4), etc. Similarly for 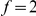, 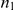 can be only 1,2 or 3, as reactions among the two food set molecules 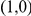 and 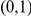 can only produce three molecules 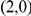, 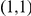 and 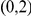.

#### Algorithm 5: Incremental, random construction of a sequence of reaction sets with partial overlap

In this algorithm we decide on a sequence of lengths, 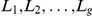, and generate an extremal ACS, denoted 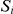, of length 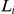 using Algorithm 3 for each 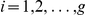. For each 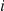 a different random number seed is used to initialize Algorithm 3. The union of the 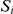
's constitutes the set of reactions in the catalyzed chemistry.

### C. Computer programs

The chemistries, spontaneous and catalyzed (including the ACSs mentioned above), are generated in C programs which are available from the authors upon request. These programs further use the CVODE library to do the numerical integration of the differential equations and also generate the inputs files required for XPPAUT, Octave and Mathematica.

## Supporting Information

Figure S1
**Pictorial representation of nested ACSs with** 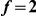 **.** Each of the three figures shows two nested ACSs. The smaller ACS in each figure is ACS(2,2), defined by Eqs. (0) in the main text. The larger ACS is different and is given by (A) ACS(5,3)(a), (B) ACS(5,3)(b), and, (C) ACS(5,3)(c), defined in the main text below Eqs. (13). The notation is the same as in Figs. 3 and 9 of the main text.(TIF)

Table S1 **List of reactions and their catalysts in ACS(8,10) referred in** **Fig. 5** **.**
(PDF)

Table S2 **List of reactions and their catalysts in ACS441 referred in** **Fig. 13** **.**
(PDF)

Table S3 **List of reactions and their catalysts in ACS(36,28) referred in** **Fig. 14** **.**
(PDF)

Table S4 **List of reactions and their catalysts in ACS(18,27) referred** **Fig. 15** **.**
(PDF)

Appendix S1
**Details of the general model and explicit examples for** 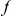 **= 1 and 2.**
(PDF)

Appendix S2

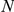
 **-independence of results at large** 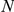 **.**
(PDF)

Appendix S3
**Dimensionless rate equations and dependence of** 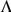 **on dimensionless parameters.**
(PDF)

## References

[1] Miller SL (1953). A production of amino acids under possible primitive earth conditions.. Science.

[2] Miller SL, Urey HC (1959). Organic compound synthesis on the primitive earth.. Science.

[3] Ricardo A, Carrigan MA, Olcott AN, Benner SA (2004). Borate minerals stabilize ribose.. Science.

[4] Powner MW, Gerland B, Sutherland JD (2009). Synthesis of activated pyrimidine ribonucleotides in prebiotically plausible conditions.. Nature.

[5] Parker ET, Cleaves HJ, Dworkin JP, Glavin DP, Callahan M (2011). Primordial synthesis of amines and amino acids in a 1958 Miller H2S-rich spark discharge experiment.. Proceedings of the National Academy of Sciences (USA).

[6] Rode BM (1999). Peptides and the origin of life.. Peptides.

[7] Ferris JP, Joshi PC, Wang K, Miyakawa S, Huang W (2004). Catalysis in prebiotic chemistry: Application to the synthesis of RNA oligomers.. Advances in Space Research.

[8] Orgel LE (2004). Prebiotic chemistry and the origin of the RNA world.. Critical Reviews in Biochemistry and Molecular Biology.

[9] Budin I, Szostak JW (2010). Expanding roles for diverse physical phenomena during the origin of life.. Annual Review of Biophysics.

[10] Barbas CF (2008). Organocatalysis lost: Modern chemistry, ancient chemistry, and an unseen biosynthetic apparatus.. Angewandte Chemie International Edition.

[11] MacMillan DWC (2008). The advent and development of organocatalysis.. Nature.

[12] Bertelsen S, Jorgensen KA (2009). Organocatalysis–after the gold rush.. Chemical Society Reviews.

[13] Severin K, Lee DH, Kennan AJ, Ghadiri MR (1997). A synthetic peptide ligase.. Nature.

[14] Brack A (2007). From interstellar amino acids to prebiotic catalytic peptides: A review.. Chemistry & Biodiversity.

[15] Cech TR, Bass BL (1986). Biological catalysis by RNA.. Annual Review of Biochemistry.

[16] Symons RH (1992). Small catalytic RNAs.. Annual Review of Biochemistry.

[17] Chen X, Li N, Ellington AD (2007). Ribozyme catalysis of metabolism in the RNA world.. Chemistry & Biodiversity.

[18] Srinivasan V, Morowitz HJ (2009). Analysis of the intermediary metabolism of a reductive chemoautotroph.. The Biological Bulletin.

[19] Eigen M (1971). Selforganization of matter and the evolution of biological macromolecules.. Die Naturwissenschaften.

[20] Kauffman SA (1971). Cellular homeostasis, epigenesis and replication in randomly aggregated macromolecular systems.. Journal of Cybernetics.

[21] Rössler OE (1971). A system theoretic model for biogenesis. Zeitschrift für Naturforschung.. Teil B: Chemie, Biochemie, Biophysik, Biologie.

[22] Farmer JD, Kauffman SA, Packard NH (1986). Autocatalytic replication of polymers.. Physica D: Nonlinear Phenomena.

[23] Bagley RJ, Farmer JD, Langton CG, Taylor CE, Farmer JD, Rasmussen S (1991). Spontaneous emergence of a metabolism..

[24] Bagley RJ, Farmer JD, Fontana W, Langton CG, Taylor CE, Farmer JD, Rasmussen S (1991). Evolution of a metabolism..

[25] Stadler P, Fontana W, Miller J (1993). Random catalytic reaction networks.. Physica D: Nonlinear Phenomena.

[26] Kauffman SA (1993). The origins of order: Self-organization and selection in evolution.

[27] Fontana W, Buss LW (1994). What would be conserved if “the tape were played twice”?. Proceedings of the National Academy of Sciences (USA).

[28] Jain S, Krishna S (1998). Autocatalytic sets and the growth of complexity in an evolutionary model.. Physical Review Letters.

[29] Jain S, Krishna S (2001). A model for the emergence of cooperation, interdependence, and structure in evolving networks.. Proceedings of the National Academy of Sciences (USA).

[30] Hanel R, Kauffman S, Thurner S (2005). Phase transition in random catalytic networks.. Physical Review E.

[31] Piedrafita G, Montero F, Morán F, Cárdenas ML, Cornish-Bowden A (2010). A simple selfmaintaining metabolic system: Robustness, autocatalysis, bistability.. PLoS Computational Biology.

[32] Eigen M, Schuster P (1977). The hypercycle: A principle of natural self-organizsation. Part A: Emergence of the hypercycle.. Die Naturwissenschaften.

[33] Szathmáry E (2006). The origin of replicators and reproducers.. Philosophical Transactions of the Royal Society B.

[34] Ohtsuki H, Nowak MA (2009). Prelife catalysts and replicators.. Proceedings of the Royal Society B.

[35] Segré D, Ben-Eli D, Lancet D (2000). Compositional genomes: prebiotic information transfer in mutually catalytic noncovalent assemblies.. Proceedings of the National Academy of Sciences (USA).

[36] Furusawa C, Kaneko K (2006). Evolutionary origin of power-laws in a biochemical reaction network: Embedding the distribution of abundance into topology.. Physical Review E.

[37] Carletti T, Serra R, Poli I, Villani M, Filisetti A (2008). Sufficient conditions for emergent synchronization in protocell models.. Journal of Theoretical Biology.

[38] Kamimura A, Kaneko K (2010). Reproduction of a protocell by replication of a minority molecule in a catalytic reaction network.. Physical Review Letters.

[39] Bagley RJ, Farmer JD, Kauffman SA, Packard NH, Perelson AS (1989). Modeling adaptive biological systems.. Bio Systems.

[40] Wächtershäuser G (1990). Evolution of the first metabolic cycles.. Proceedings of the National Academy of Sciences (USA).

[41] Morowitz HJ, Kostelnik JD, Yang J, Cody GD (2000). The origin of intermediary metabolism.. Proceedings of the National Academy of Sciences (USA).

[42] Hindmarsh AC, Brown PN, Grant KE, Lee SL, Serban R (2005). SUNDIALS: Suite of nonlinear and differential/algebraic equation solvers.. ACM Transactions on Mathematical Software.

[43] Ermentrout B (2002). Simulating, Analyzing, and Animating Dynamical Systems: A Guide to XPPAUT for Researchers and Students.

[44] Octave.. http://www.gnu.org/software/octave.

[45] Wolfram Research, Inc. (2008). Mathematica, Version 7.0..

[46] Craciun G, Tang Y, Feinberg M (2006). Understanding bistability in complex enzyme-driven reaction networks.. Proceedings of the National Academy of Sciences (USA).

[47] Angeli D, Hirsch MW, Sontag ED (2009). Attractors in coherent systems of differential equations.. Journal of Differential Equations.

[48] Craciun G, Pantea C, Sontag ED, Koeppl H, Setti G, di Bernardo M, Densmore D (2011). Graph-Theoretic Analysis of Multistability and Monotonicity for Biochemical Reaction Networks.. Design and Analysis of Biomolecular Circuits: Engineering Approaches to Systems and Synthetic Biology.

[49] Wilhelm T (2007). Analysis of structures causing instabilities.. Physical Review E.

[50] Ramakrishnan N, Bhalla US (2008). Memory switches in chemical reaction space.. PLoS computational biology.

[51] Copley SD, Smith E, Morowitz HJ (2007). The origin of the RNA world: co-evolution of genes and metabolism.. Bioorganic Chemistry.

[52] Powner MW, Sutherland JD (2011). Prebiotic chemistry: A new modus operandi.. Philosophical Transactions of the Royal Society B.

[53] Morowitz HJ (1999). A theory of biochemical organization, metabolic pathways, and evolution.. Complexity.

[54] Steel M (2000). The emergence of a self-catalysing structure in abstract origin-of-life models.. Applied Mathematics Letters.

[55] Hordijk W, Kauffman SA, Steel M (2011). Required levels of catalysis for emergence of autocatalytic sets in models of chemical reaction systems.. International journal of molecular sciences.

[56] Kun A, Papp B, Szathmáry E (2008). Computational identification of obligatorily autocatalytic replicators embedded in metabolic networks.. Genome biology.

[57] Orgel LE (2000). Self-organizing biochemical cycles.. Proceedings of the National Academy of Sciences (USA).

[58] Orgel LE (2008). The implausibility of metabolic cycles on the prebiotic earth.. PLoS biology.

[59] Wales DJ (2004). Energy landscapes: Applications to clusters, biomolecules and glasses.

