# Supplementary figures and images for: The Origin of Large Molecules in Primordial Autocatalytic Reaction Networks
Source: PLoS One. 2012 Jan 4;7(1):e29546. doi: 10.1371/journal.pone.0029546 (PMC3251582; doi:10.1371/journal.pone.0029546)

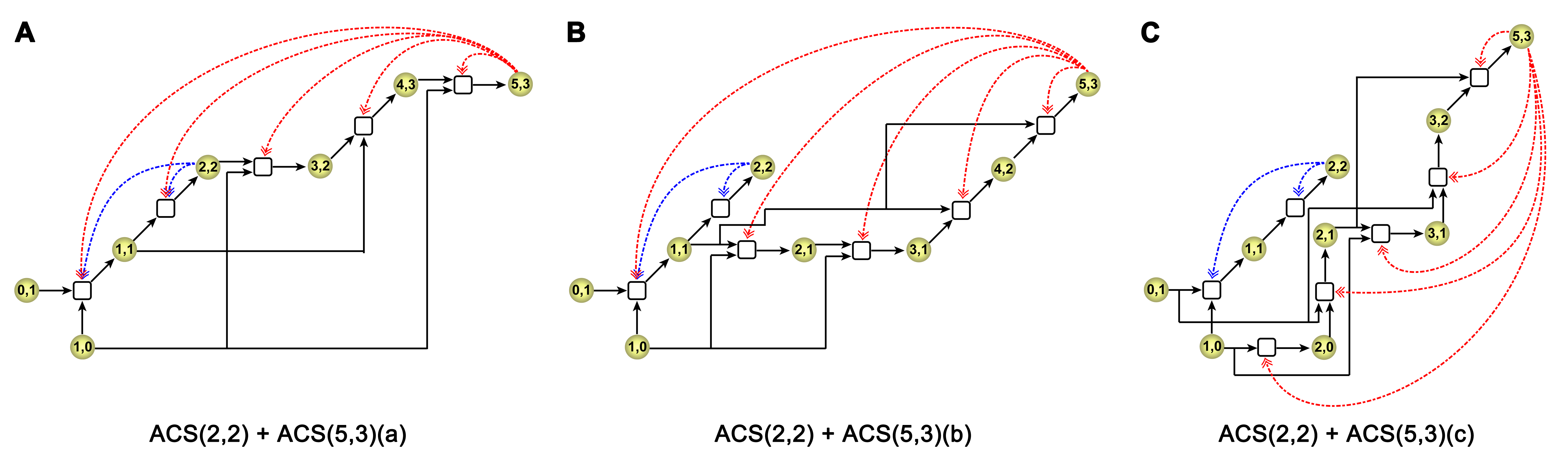

Supplement: Figure S1 — Pictorial representation of nested ACSs with . Each of the three figures shows two nested ACSs. The smaller ACS in each figure is ACS(2,2), defined by Eqs. (0) in the main text. The larger ACS is different and is given by (A) ACS(5,3)(a), (B) ACS(5,3)(b), and, (C) ACS(5,3)(c), defined in the main text below Eqs. (13). The notation is the same as in Figs. 3 and 9 of the main text. (TIF) [file pone.0029546.s001.tif]
