# Supplementary material for: The Origin of Large Molecules in Primordial Autocatalytic Reaction Networks
Source: PLoS One. 2012 Jan 4;7(1):e29546. doi: 10.1371/journal.pone.0029546 (PMC3251582; doi:10.1371/journal.pone.0029546)
Supplement: Table S1 — List of reactions and their catalysts in ACS(8,10) referred in Fig. 5 . (PDF) [file pone.0029546.s002.pdf]

The origin of large molecules in primordial autocatalytic reaction networks

Varun Giri and Sanjay Jain

Supporting Information: Table S1

List of reactions and their catalysts in ACS(8,10) (referred in Fig. 5 of main text)

**Reaction**	**Catalyst**
$(0,1) + (1,0) \rightleftharpoons (1,1)$	$(1,3)$
$(1,0) + (1,0) \rightleftharpoons (2,0)$	$(3,6)$
$(0,1) + (1,1) \rightleftharpoons (1,2)$	$(5,6)$
$(0,1) + (1,2) \rightleftharpoons (1,3)$	$(8,10)$
$(1,1) + (1,2) \rightleftharpoons (2,3)$	$(1,3)$
$(1,3) + (2,3) \rightleftharpoons (3,6)$	$(1,2)$
$(2,0) + (3,6) \rightleftharpoons (5,6)$	$(2,3)$
$(2,3) + (5,6) \rightleftharpoons (7,9)$	$(3,6)$
$(1,1) + (7,9) \rightleftharpoons (8,10)$	$(2,3)$

Note that this catalyzed chemistry forms an ACS. Each catalyst in the above list gets produced by a reaction of this chemistry. Also, every reactant is either a member of the set $\mathcal{F}$ or gets produced in this chemistry.
