# Supplementary material for: The Origin of Large Molecules in Primordial Autocatalytic Reaction Networks
Source: PLoS One. 2012 Jan 4;7(1):e29546. doi: 10.1371/journal.pone.0029546 (PMC3251582; doi:10.1371/journal.pone.0029546)
Supplement: Table S2 — List of reactions and their catalysts in ACS441 referred in Fig. 13 . (PDF) [file pone.0029546.s003.pdf]

The origin of large molecules in primordial autocatalytic reaction networks

Varun Giri and Sanjay Jain

Supporting Information: Table S2

List of reactions and their catalysts in ACS441 (referred in Fig. 13 of main text)

The table lists all the reactions with their respective catalysts in the example of a catalyzed chemistry, quoted in the main text, containing a cascade of nested ACSs for $f = 1$ generated using Algorithm 4. The steady state concentrations for this chemistry are displayed in Fig. 13. This chemistry was generated with $g = 15$ and $n_1 = 1, n_2 = n_3 = \ldots = n_{15} = 2$.

Note that a general molecule for the $f = 1$ case, represented by $\mathrm{A}(n)$ in the main text, has been represented here by n for brevity.

The molecules in various generations are as follows: $P_0 = \{1\}$, $P_1 = \{2\}$, $P_2 = \{3, 4\}$, $P_3 = \{6, 7\}$, $P_4 = \{10, 12\}$, $P_5 = \{17, 18\}$, $P_6 = \{20, 24\}$, $P_7 = \{25, 48\}$, $P_8 = \{52, 66\}$, $P_9 = \{67, 69\}$, $P_{10} = \{77, 84\}$, $P_{11} = \{144, 168\}$, $P_{12} = \{221, 288\}$, $P_{13} = \{305, 336\}$, $P_{14} = \{372, 389\}$, $P_{15} = \{397, 441\}$.

The catalyst for a reaction listed under generation P_k is added at step k of algorithm. It is apparent from the reaction table that the ACSs are maximally overlapping, *i.e.*, any ACS of generation k contains all the reactions of generation $k - 1$.

**Reaction**	**Catalyst added in generation**														
	P_1	P_2	P_3	P_4	P_5	P_6	P_7	P_8	P_9	P_{10}	P_{11}	P_{12}	P_{13}	P_{14}	P_{15}
$1 + 1 \rightleftharpoons 2$	2	3	6	10	17	24	48	52	69	84	144	288	336	372	397
$1 + 2 \rightleftharpoons 3$		3	7	10	18	20	25	52	67	77	168	221	305	372	397
$2 + 2 \rightleftharpoons 4$		4	7	10	18	20	25	66	67	77	168	221	336	372	397
$3 + 3 \rightleftharpoons 6$			6	12	17	24	25	66	67	84	144	221	336	372	441
$3 + 4 \rightleftharpoons 7$			7	12	18	24	48	52	67	84	144	221	336	372	397
$4 + 6 \rightleftharpoons 10$				10	18	24	48	52	69	77	168	288	336	389	397
$6 + 6 \rightleftharpoons 12$				12	17	20	48	66	69	77	144	221	305	372	441
$7 + 10 \rightleftharpoons 17$					17	24	25	52	69	84	168	221	336	389	441
$6 + 12 \rightleftharpoons 18$					18	20	48	66	69	77	168	288	336	372	441
$2 + 18 \rightleftharpoons 20$						20	48	52	69	84	144	221	336	389	441
$7 + 17 \rightleftharpoons 24$						20	25	66	69	77	168	221	336	372	397
$1 + 24 \rightleftharpoons 25$							48	52	69	84	144	288	305	372	441
$24 + 24 \rightleftharpoons 48$							48	66	67	77	144	221	305	389	441
$4 + 48 \rightleftharpoons 52$								66	69	84	168	221	336	372	397

continued on next page . . .

... continued from previous page

**Reaction**	**Catalyst in generation**														
	P_1	P_2	P_3	P_4	P_5	P_6	P_7	P_8	P_9	P_{10}	P_{11}	P_{12}	P_{13}	P_{14}	P_{15}
$18 + 48 \rightleftharpoons 66$								66	69	84	144	288	336	389	441
$1 + 66 \rightleftharpoons 67$									67	77	168	221	336	389	441
$3 + 66 \rightleftharpoons 69$									67	84	168	221	305	372	441
$10 + 67 \rightleftharpoons 77$										77	168	288	305	372	441
$17 + 67 \rightleftharpoons 84$										77	144	221	305	389	397
$67 + 77 \rightleftharpoons 144$											144	288	305	389	397
$84 + 84 \rightleftharpoons 168$											144	221	336	389	441
$77 + 144 \rightleftharpoons 221$												221	305	372	397
$144 + 144 \rightleftharpoons 288$												221	336	372	441
$17 + 288 \rightleftharpoons 305$													305	372	441
$48 + 288 \rightleftharpoons 336$													336	389	397
$67 + 305 \rightleftharpoons 372$														389	397
$84 + 305 \rightleftharpoons 389$														389	397
$25 + 372 \rightleftharpoons 397$															441
$69 + 372 \rightleftharpoons 441$															397
