# Supplementary material for: The Origin of Large Molecules in Primordial Autocatalytic Reaction Networks
Source: PLoS One. 2012 Jan 4;7(1):e29546. doi: 10.1371/journal.pone.0029546 (PMC3251582; doi:10.1371/journal.pone.0029546)
Supplement: Table S3 — List of reactions and their catalysts in ACS(36,28) referred in Fig. 14 . (PDF) [file pone.0029546.s004.pdf]

**The origin of large molecules in primordial autocatalytic reaction networks**

**Varun Giri and Sanjay Jain**

# Supporting Information: Table S3

# List of reactions and their catalysts in ACS(36,28) (referred in Fig. 14 of main text)

The table lists all the reactions with their respective catalysts in the example of a catalyzed chemistry, quoted in the main text, containing a cascade of nested ACSs for $f = 2$ generated using Algorithm 4. The steady state concentrations for this chemistry are displayed in Fig. 14. This chemistry was generated with $g = 7$ and $n_k = 3$.

The molecules in various generations are as follows: $P_0 = \{(1,0),(0,1)\}$, $P_1 = \{(1,1),(0,2),(2,0)\}$, $P_2 = \{(1,3),(2,2),(3,0)\}$, $P_3 = \{(2,6),(2,3),(5,2)\}$, $P_4 = \{(4,6),(7,4),(7,8)\}$, $P_5 = \{(8,12),(6,12),(14,8)\}$, $P_6 = \{(14,24),(22,20),(11,12)\}$, $P_7 = \{(29,28),(36,28),(24,26)\}$.

The catalyst for a reaction listed under generation $P_k$ is added at step $k$ of algorithm. It is apparent from the reaction table that the ACSs are maximally overlapping, *i.e.*, any ACS of generation $k$ contains all the reactions of generation $k-1$.

| **Reaction** | **Catalyst added in generation** | | | | | | |
|---|---|---|---|---|---|---|---|
| | $P_1$ | $P_2$ | $P_3$ | $P_4$ | $P_5$ | $P_6$ | $P_7$ |
| $(0,1)+(0,1) \rightleftharpoons (0,2)$ | $(1,1)$ | $(1,3)$ | $(2,3)$ | $(7,4)$ | $(8,12)$ | $(11,12)$ | $(29,28)$ |
| $(0,1)+(1,0) \rightleftharpoons (1,1)$ | $(0,2)$ | $(1,3)$ | $(2,6)$ | $(4,6)$ | $(14,8)$ | $(22,20)$ | $(36,28)$ |
| $(1,0)+(1,0) \rightleftharpoons (2,0)$ | $(1,1)$ | $(3,0)$ | $(5,2)$ | $(7,4)$ | $(14,8)$ | $(14,24)$ | $(29,28)$ |
| $(1,0)+(2,0) \rightleftharpoons (3,0)$ | | $(2,2)$ | $(5,2)$ | $(4,6)$ | $(14,8)$ | $(14,24)$ | $(24,26)$ |
| $(1,1)+(0,2) \rightleftharpoons (1,3)$ | | $(1,3)$ | $(2,6)$ | $(7,8)$ | $(8,12)$ | $(14,24)$ | $(24,26)$ |
| $(0,2)+(2,0) \rightleftharpoons (2,2)$ | | $(1,3)$ | $(2,6)$ | $(7,8)$ | $(6,12)$ | $(22,20)$ | $(29,28)$ |
| $(0,1)+(2,2) \rightleftharpoons (2,3)$ | | | $(2,6)$ | $(7,4)$ | $(14,8)$ | $(22,20)$ | $(24,26)$ |
| $(3,0)+(2,2) \rightleftharpoons (5,2)$ | | | $(2,3)$ | $(7,8)$ | $(6,12)$ | $(11,12)$ | $(29,28)$ |
| $(1,3)+(1,3) \rightleftharpoons (2,6)$ | | | $(5,2)$ | $(7,8)$ | $(14,8)$ | $(22,20)$ | $(24,26)$ |
| $(2,0)+(2,6) \rightleftharpoons (4,6)$ | | | | $(4,6)$ | $(14,8)$ | $(22,20)$ | $(36,28)$ |
| $(2,2)+(5,2) \rightleftharpoons (7,4)$ | | | | $(7,4)$ | $(6,12)$ | $(14,24)$ | $(36,28)$ |
| $(5,2)+(2,6) \rightleftharpoons (7,8)$ | | | | $(7,4)$ | $(14,8)$ | $(14,24)$ | $(36,28)$ |
| $(2,6)+(4,6) \rightleftharpoons (6,12)$ | | | | | $(14,8)$ | $(11,12)$ | $(29,28)$ |
| $(4,6)+(4,6) \rightleftharpoons (8,12)$ | | | | | $(14,8)$ | $(22,20)$ | $(36,28)$ |
| $(7,4)+(7,4) \rightleftharpoons (14,8)$ | | | | | $(8,12)$ | $(11,12)$ | $(36,28)$ |
| $(3,0)+(8,12) \rightleftharpoons (11,12)$ | | | | | | $(22,20)$ | $(36,28)$ |

*continued on next page ...*

*...continued from previous page*

| **Reaction** | **Catalyst in generation** | | | | | | |
|---|---|---|---|---|---|---|---|
| | $P_1$ | $P_2$ | $P_3$ | $P_4$ | $P_5$ | $P_6$ | $P_7$ |
| $(8,12)+(6,12)\rightleftharpoons(14,24)$ | | | | | | $(22,20)$ | $(24,26)$ |
| $(14,8)+(8,12)\rightleftharpoons(22,20)$ | | | | | | $(11,12)$ | $(29,28)$ |
| $(2,6)+(22,20)\rightleftharpoons(24,26)$ | | | | | | | $(29,28)$ |
| $(7,8)+(22,20)\rightleftharpoons(29,28)$ | | | | | | | $(29,28)$ |
| $(14,8)+(22,20)\rightleftharpoons(36,28)$ | | | | | | | $(29,28)$ |
