# Supplementary material for: The Origin of Large Molecules in Primordial Autocatalytic Reaction Networks
Source: PLoS One. 2012 Jan 4;7(1):e29546. doi: 10.1371/journal.pone.0029546 (PMC3251582; doi:10.1371/journal.pone.0029546)
Supplement: Table S4 — List of reactions and their catalysts in ACS(18,27) referred Fig. 15 . (PDF) [file pone.0029546.s005.pdf]

The origin of large molecules in primordial autocatalytic reaction networks

Varun Giri and Sanjay Jain

Supporting Information: Table S4

List of reactions and their catalysts in ACS(18,27) (referred in Fig. 15 of main text)

The table lists all the reactions with their respective catalysts and catalytic strengths in the example of a catalyzed chemistry, quoted in the main text, containing a cascade of partially overlapping ACSs for $f = 2$ generated using Algorithm 5. The steady state concentrations for this chemistry are displayed in Fig. 15.

The catalyzed chemistry contains 10 generations of ACSs of lengths 3, 6, 10, 15, 19, 25, 30, 35, 40, and 45.

TABLE S4a: **List of reactions in the ACSs of different length and their catalysts.** ACSs of increasing length (using Algorithm 5) are added in the chemistry. The length of the ACS and the catalytic strength of the catalyst are mentioned in the table.

Reaction	Catalyst
**Generation 1 of length 3** ($\kappa = 1000$)	
$(1,0) + (1,0) \rightleftharpoons (2,0)$	$(3,0)$
$(2,0) + (1,0) \rightleftharpoons (3,0)$	$(3,0)$
**Generation 2 of length 6** ($\kappa = 2000$)	
$(1,0) + (1,0) \rightleftharpoons (2,0)$	$(4,2)$
$(0,1) + (2,0) \rightleftharpoons (2,1)$	$(4,2)$
$(2,1) + (0,1) \rightleftharpoons (2,2)$	$(4,2)$
$(2,1) + (2,1) \rightleftharpoons (4,2)$	$(4,2)$
**Generation 3 of length 10** ($\kappa = 4000$)	
$(1,0) + (0,1) \rightleftharpoons (1,1)$	$(4,6)$
$(1,1) + (1,0) \rightleftharpoons (2,1)$	$(4,6)$
$(1,1) + (1,1) \rightleftharpoons (2,2)$	$(4,6)$
$(0,1) + (2,2) \rightleftharpoons (2,3)$	$(4,6)$
$(2,3) + (2,3) \rightleftharpoons (4,6)$	$(4,6)$
**Generation 4 of length 15** ($\kappa = 7000$)	
$(0,1) + (0,1) \rightleftharpoons (0,2)$	$(7,8)$
$(0,2) + (1,0) \rightleftharpoons (1,2)$	$(7,8)$
$(1,2) + (1,0) \rightleftharpoons (2,2)$	$(7,8)$
$(2,2) + (2,2) \rightleftharpoons (4,4)$	$(7,8)$

continued on next page ...

**Reaction**	**Catalyst**
$(1,2)+(4,4) \rightleftharpoons (5,6)$	$(7,8)$
$(5,6)+(1,2) \rightleftharpoons (6,8)$	$(7,8)$
$(5,6)+(2,2) \rightleftharpoons (7,8)$	$(7,8)$
**Generation 5 of length 19** ($\kappa = 10000$)	
$(0,1)+(1,0) \rightleftharpoons (1,1)$	$(7,12)$
$(1,1)+(0,1) \rightleftharpoons (1,2)$	$(7,12)$
$(1,2)+(1,2) \rightleftharpoons (2,4)$	$(7,12)$
$(1,1)+(2,4) \rightleftharpoons (3,5)$	$(7,12)$
$(2,4)+(3,5) \rightleftharpoons (5,9)$	$(7,12)$
$(1,1)+(5,9) \rightleftharpoons (6,10)$	$(7,12)$
$(1,2)+(6,10) \rightleftharpoons (7,12)$	$(7,12)$
**Generation 6 of length 25** ($\kappa = 15000$)	
$(0,1)+(1,0) \rightleftharpoons (1,1)$	$(12,13)$
$(1,1)+(1,0) \rightleftharpoons (2,1)$	$(12,13)$
$(2,1)+(0,1) \rightleftharpoons (2,2)$	$(12,13)$
$(2,2)+(0,1) \rightleftharpoons (2,3)$	$(12,13)$
$(1,0)+(2,3) \rightleftharpoons (3,3)$	$(12,13)$
$(2,1)+(3,3) \rightleftharpoons (5,4)$	$(12,13)$
$(2,3)+(5,4) \rightleftharpoons (7,7)$	$(12,13)$
$(7,7)+(2,2) \rightleftharpoons (9,9)$	$(12,13)$
$(7,7)+(3,3) \rightleftharpoons (10,10)$	$(12,13)$
$(9,9)+(2,3) \rightleftharpoons (11,12)$	$(12,13)$
$(11,12)+(1,1) \rightleftharpoons (12,13)$	$(12,13)$
**Generation 7 of length 30** ($\kappa = 20000$)	
$(0,1)+(1,0) \rightleftharpoons (1,1)$	$(13,17)$
$(1,1)+(0,1) \rightleftharpoons (1,2)$	$(13,17)$
$(1,2)+(1,1) \rightleftharpoons (2,3)$	$(13,17)$
$(1,0)+(2,3) \rightleftharpoons (3,3)$	$(13,17)$
$(3,3)+(1,1) \rightleftharpoons (4,4)$	$(13,17)$
$(3,3)+(1,2) \rightleftharpoons (4,5)$	$(13,17)$
$(2,3)+(4,5) \rightleftharpoons (6,8)$	$(13,17)$
$(2,3)+(6,8) \rightleftharpoons (8,11)$	$(13,17)$
$(6,8)+(4,4) \rightleftharpoons (10,12)$	$(13,17)$
$(10,12)+(2,3) \rightleftharpoons (12,15)$	$(13,17)$
$(12,15)+(1,2) \rightleftharpoons (13,17)$	$(13,17)$
**Generation 8 of length 35** ($\kappa = 27000$)	

continued on next page ...

TABLE S4a *... continued from previous page*

**Reaction**	**Catalyst**
$(1,0)+(0,1) \rightleftharpoons (1,1)$	$(15,20)$
$(0,1)+(1,1) \rightleftharpoons (1,2)$	$(15,20)$
$(1,1)+(1,2) \rightleftharpoons (2,3)$	$(15,20)$
$(1,2)+(2,3) \rightleftharpoons (3,5)$	$(15,20)$
$(3,5)+(1,1) \rightleftharpoons (4,6)$	$(15,20)$
$(1,1)+(4,6) \rightleftharpoons (5,7)$	$(15,20)$
$(3,5)+(4,6) \rightleftharpoons (7,11)$	$(15,20)$
$(7,11)+(4,6) \rightleftharpoons (11,17)$	$(15,20)$
$(1,0)+(11,17) \rightleftharpoons (12,17)$	$(15,20)$
$(1,1)+(12,17) \rightleftharpoons (13,18)$	$(15,20)$
$(13,18)+(1,2) \rightleftharpoons (14,20)$	$(15,20)$
$(1,0)+(14,20) \rightleftharpoons (15,20)$	$(15,20)$
**Generation 9 of length 40 ($\kappa = 35000$)**	
$(0,1)+(0,1) \rightleftharpoons (0,2)$	$(14,26)$
$(1,0)+(0,2) \rightleftharpoons (1,2)$	$(14,26)$
$(1,2)+(1,2) \rightleftharpoons (2,4)$	$(14,26)$
$(1,2)+(2,4) \rightleftharpoons (3,6)$	$(14,26)$
$(3,6)+(1,0) \rightleftharpoons (4,6)$	$(14,26)$
$(3,6)+(2,4) \rightleftharpoons (5,10)$	$(14,26)$
$(5,10)+(1,2) \rightleftharpoons (6,12)$	$(14,26)$
$(6,12)+(2,4) \rightleftharpoons (8,16)$	$(14,26)$
$(2,4)+(8,16) \rightleftharpoons (10,20)$	$(14,26)$
$(0,1)+(10,20) \rightleftharpoons (10,21)$	$(14,26)$
$(10,21)+(0,2) \rightleftharpoons (10,23)$	$(14,26)$
$(0,2)+(10,23) \rightleftharpoons (10,25)$	$(14,26)$
$(10,23)+(1,2) \rightleftharpoons (11,25)$	$(14,26)$
$(8,16)+(5,10) \rightleftharpoons (13,26)$	$(14,26)$
$(4,6)+(10,20) \rightleftharpoons (14,26)$	$(14,26)$
**Generation 10 of length 45 ($\kappa = 50000$)**	
$(0,1)+(1,0) \rightleftharpoons (1,1)$	$(18,27)$
$(0,1)+(1,1) \rightleftharpoons (1,2)$	$(18,27)$
$(1,2)+(1,0) \rightleftharpoons (2,2)$	$(18,27)$
$(1,2)+(1,1) \rightleftharpoons (2,3)$	$(18,27)$
$(1,2)+(2,2) \rightleftharpoons (3,4)$	$(18,27)$
$(2,3)+(2,3) \rightleftharpoons (4,6)$	$(18,27)$
$(4,6)+(1,1) \rightleftharpoons (5,7)$	$(18,27)$

continued on next page ...

TABLE S4a *... continued from previous page*

**Reaction**	**Catalyst**
$(4,6) + (3,4) \rightleftharpoons (7,10)$	$(18,27)$
$(7,10) + (7,10) \rightleftharpoons (14,20)$	$(18,27)$
$(0,1) + (14,20) \rightleftharpoons (14,21)$	$(18,27)$
$(14,21) + (2,2) \rightleftharpoons (16,23)$	$(18,27)$
$(2,3) + (16,23) \rightleftharpoons (18,26)$	$(18,27)$
$(14,21) + (4,6) \rightleftharpoons (18,27)$	$(18,27)$

TABLE S4b: **List of reactions in the catalyzed chemistry and their catalysts.** The table lists all the reactions that are part of the catalyzed chemistry with all its catalysts. The catalysts that belong to different generations (G_1 to G_{10}) have been separated in different columns. It is easy to see from this table the amount of overlap between any two nested ACSs. For example, between the ACSs G_5 and G_6 which contain, respectively, 7 and 11 reactions, only one is common.

**Reaction**	**Catalyst**									
	G_1	G_2	G_3	G_4	G_5	G_6	G_7	G_8	G_9	G_{10}
$(0,1) + (0,1) \rightleftharpoons (0,2)$				(7,8)					(14,26)	
$(1,0) + (0,1) \rightleftharpoons (1,1)$			(4,6)		(7,12)	(12,13)	(13,17)	(15,20)		(18,27)
$(1,0) + (1,0) \rightleftharpoons (2,0)$	(3,0)	(4,2)								
$(0,2) + (1,0) \rightleftharpoons (1,2)$				(7,8)					(14,26)	
$(1,1) + (0,1) \rightleftharpoons (1,2)$					(7,12)		(13,17)	(15,20)		(18,27)
$(0,1) + (2,0) \rightleftharpoons (2,1)$		(4,2)								
$(1,1) + (1,0) \rightleftharpoons (2,1)$			(4,6)			(12,13)				
$(2,0) + (1,0) \rightleftharpoons (3,0)$	(3,0)									
$(2,1) + (0,1) \rightleftharpoons (2,2)$		(4,2)				(12,13)				
$(1,1) + (1,1) \rightleftharpoons (2,2)$			(4,6)							
$(1,2) + (1,0) \rightleftharpoons (2,2)$				(7,8)						(18,27)
$(0,1) + (2,2) \rightleftharpoons (2,3)$			(4,6)			(12,13)				
$(1,2) + (1,1) \rightleftharpoons (2,3)$							(13,17)	(15,20)		(18,27)
$(1,2) + (1,2) \rightleftharpoons (2,4)$					(7,12)				(14,26)	
$(1,0) + (2,3) \rightleftharpoons (3,3)$						(12,13)	(13,17)			
$(2,1) + (2,1) \rightleftharpoons (4,2)$		(4,2)								
$(1,2) + (2,2) \rightleftharpoons (3,4)$										(18,27)
$(1,1) + (2,4) \rightleftharpoons (3,5)$					(7,12)					
$(1,2) + (2,3) \rightleftharpoons (3,5)$								(15,20)		
$(2,2) + (2,2) \rightleftharpoons (4,4)$				(7,8)						

continued on next page ...

TABLE S4b *... continued from previous page*

**Reaction**	**Catalyst**									
	G_1	G_2	G_3	G_4	G_5	G_6	G_7	G_8	G_9	G_{10}
$(3,3) + (1,1) \rightleftharpoons (4,4)$							(13,17)			
$(1,2) + (2,4) \rightleftharpoons (3,6)$									(14,26)	
$(3,3) + (1,2) \rightleftharpoons (4,5)$							(13,17)			
$(2,1) + (3,3) \rightleftharpoons (5,4)$						(12,13)				
$(2,3) + (2,3) \rightleftharpoons (4,6)$			(4,6)							(18,27)
$(3,5) + (1,1) \rightleftharpoons (4,6)$								(15,20)		
$(3,6) + (1,0) \rightleftharpoons (4,6)$									(14,26)	
$(1,2) + (4,4) \rightleftharpoons (5,6)$				(7,8)						
$(1,1) + (4,6) \rightleftharpoons (5,7)$								(15,20)		(18,27)
$(2,4) + (3,5) \rightleftharpoons (5,9)$					(7,12)					
$(5,6) + (1,2) \rightleftharpoons (6,8)$				(7,8)						
$(2,3) + (4,5) \rightleftharpoons (6,8)$							(13,17)			
$(2,3) + (5,4) \rightleftharpoons (7,7)$						(12,13)				
$(3,6) + (2,4) \rightleftharpoons (5,10)$									(14,26)	
$(5,6) + (2,2) \rightleftharpoons (7,8)$				(7,8)						
$(1,1) + (5,9) \rightleftharpoons (6,10)$					(7,12)					
$(4,6) + (3,4) \rightleftharpoons (7,10)$										(18,27)
$(5,10) + (1,2) \rightleftharpoons (6,12)$									(14,26)	
$(3,5) + (4,6) \rightleftharpoons (7,11)$								(15,20)		
$(7,7) + (2,2) \rightleftharpoons (9,9)$						(12,13)				
$(1,2) + (6,10) \rightleftharpoons (7,12)$					(7,12)					
$(2,3) + (6,8) \rightleftharpoons (8,11)$							(13,17)			
$(7,7) + (3,3) \rightleftharpoons (10,10)$						(12,13)				
$(6,8) + (4,4) \rightleftharpoons (10,12)$							(13,17)			
$(9,9) + (2,3) \rightleftharpoons (11,12)$						(12,13)				
$(6,12) + (2,4) \rightleftharpoons (8,16)$									(14,26)	
$(11,12) + (1,1) \rightleftharpoons (12,13)$						(12,13)				
$(10,12) + (2,3) \rightleftharpoons (12,15)$							(13,17)			
$(7,11) + (4,6) \rightleftharpoons (11,17)$								(15,20)		
$(1,0) + (11,17) \rightleftharpoons (12,17)$								(15,20)		
$(2,4) + (8,16) \rightleftharpoons (10,20)$									(14,26)	
$(12,15) + (1,2) \rightleftharpoons (13,17)$							(13,17)			
$(0,1) + (10,20) \rightleftharpoons (10,21)$									(14,26)	
$(1,1) + (12,17) \rightleftharpoons (13,18)$								(15,20)		
$(10,21) + (0,2) \rightleftharpoons (10,23)$									(14,26)	

continued on next page ...

TABLE S4b *... continued from previous page*

Reaction	Catalyst									
	G_1	G_2	G_3	G_4	G_5	G_6	G_7	G_8	G_9	G_{10}
$(13,18)+(1,2)\rightleftharpoons(14,20)$								(15,20)		
$(7,10)+(7,10)\rightleftharpoons(14,20)$										(18,27)
$(0,2)+(10,23)\rightleftharpoons(10,25)$									(14,26)	
$(0,1)+(14,20)\rightleftharpoons(14,21)$										(18,27)
$(1,0)+(14,20)\rightleftharpoons(15,20)$								(15,20)		
$(10,23)+(1,2)\rightleftharpoons(11,25)$									(14,26)	
$(8,16)+(5,10)\rightleftharpoons(13,26)$									(14,26)	
$(14,21)+(2,2)\rightleftharpoons(16,23)$										(18,27)
$(4,6)+(10,20)\rightleftharpoons(14,26)$									(14,26)	
$(2,3)+(16,23)\rightleftharpoons(18,26)$										(18,27)
$(14,21)+(4,6)\rightleftharpoons(18,27)$										(18,27)
