# Supplementary material for: The Origin of Large Molecules in Primordial Autocatalytic Reaction Networks
Source: PLoS One. 2012 Jan 4;7(1):e29546. doi: 10.1371/journal.pone.0029546 (PMC3251582; doi:10.1371/journal.pone.0029546)
Supplement: Appendix S1 — Details of the general model and explicit examples for = 1 and 2. (PDF) [file pone.0029546.s006.pdf]

The origin of large molecules in primordial autocatalytic reaction networks

Varun Giri and Sanjay Jain

Supporting Information: Appendix S1

Details of the general model and explicit examples for f = 1 and 2

I. RATE EQUATIONS IN A GENERAL CASE

When there are f food set species, $\mathcal{F} = \{A_1, A_2, \ldots, A_f\}$, a general molecule is represented as $A = (a_1, a_2, \ldots, a_f)$, where a_i (a non-negative integer) is the number of monomers of type A_i contained in A. The 'size' or 'length' n of the molecule is defined as the total number of monomers of all types in it: $n = \sum_i a_i$. The food set molecules themselves are represented by the f-tuples $(1, 0, 0, \ldots, 0), (0, 1, 0, 0, \ldots, 0), \ldots, (0, 0, \ldots, 0, 1)$.

A general reaction pair is given by $B + C \rightleftharpoons A$, where $A = (a_1, a_2, \ldots, a_f)$, $B = (b_1, b_2, \ldots, b_f)$ and $C = (c_1, c_2, \ldots, c_f)$, with $a_i = b_i + c_i$. If x_A denotes the concentration of the molecule A, the rate equations are given by $\dot{x}_A = 0$ if $A \in \mathcal{F}$; otherwise,

$$\dot{x}_A = \sum_{(B,C)\in\mathcal{Q}_A} v_{BC} - \sum_{B, B\neq A} v_{AB} - 2v_{AA} - \phi_A x_A, \quad \text{(S1.1)}$$

where $v_{XY} = k^F_{XY} x_X x_Y - k^R_{XY} x_Z$ is the net forward flux of the reaction pair $X + Y \rightleftharpoons Z$ with forward rate constant k^F_{XY} and reverse rate constant k^R_{XY}, ϕ_A is the loss rate of A, and $\mathcal{Q}_A$ represents the set of unordered pairs of molecules which can combine together to form A ($\mathcal{Q}_A = \{(B, C) : b_i + c_i = a_i, \text{ for all } i = 1, 2, \ldots, f\}$).

II. RATE EQUATIONS FOR A CHEMISTRY WITH $f = 1$

For $f = 1$, instead of using a '1-tuple', we represent the molecules of size n by the notation $A(n)$ for clarity. Following Eq. (S1.1), rate equations for the system are given by $\dot{x}_1 = 0$ and for $n = 2, 3, \ldots$,

$$\dot{x}_n = \sum_{i\leq j, i+j=n} v_{ij} - \sum_{i=1, i\neq n}^{\infty} v_{in} - 2v_{nn} - \phi_n x_n \quad \text{(S1.2)}$$

$$= \sum_{i\leq j, i+j=n} \left(k^F_{ij} x_i x_j - k^R_{ij} x_n\right) - \sum_{i=1, i\neq n}^{\infty} \left(k^F_{in} x_i x_n - k^R_{in} x_{i+n}\right) - 2\left(k^F_{nn} x_n^2 - k^R_{nn} x_{2n}\right) - \phi_n x_n. \quad \text{(S1.3)}$$

Explicitly,

$$\begin{aligned}
\dot{x}_2 &= \left(k^F_{11}x_1^2 - k^R_{11}x_2\right) - \left(k^F_{12}x_1x_2 - k^R_{12}x_3\right) - 2\left(k^F_{22}x_2^2 - k^R_{22}x_4\right) - \left(k^F_{32}x_3x_2 - k^R_{32}x_5\right) - \ldots - \phi_2 x_2 \\
\dot{x}_3 &= \left(k^F_{12}x_1x_2 - k^R_{12}x_3\right) - \left(k^F_{13}x_1x_3 - k^R_{13}x_4\right) - \left(k^F_{23}x_2x_3 - k^R_{23}x_5\right) - 2\left(k^F_{33}x_3^2 - k^R_{33}x_6\right) - \left(k^F_{43}x_4x_3 - k^R_{43}x_7\right) \\
&\quad - \ldots - \phi_3 x_3 \\
\dot{x}_4 &= \left(k^F_{13}x_1x_3 - k^R_{13}x_4\right) + \left(k^F_{22}x_2^2 - k^R_{22}x_4\right) - \left(k^F_{14}x_1x_4 - k^R_{14}x_5\right) - \left(k^F_{24}x_2x_4 - k^R_{24}x_6\right) - \left(k^F_{34}x_3x_4 - k^R_{34}x_7\right) \\
&\quad -2\left(k^F_{44}x_4^2 - k^R_{44}x_8\right) - \left(k^F_{54}x_5x_4 - k^R_{54}x_9\right) - \ldots - \phi_4 x_4 \\
&\vdots
\end{aligned}$$

Truncation of the chemistry for numerical simulations: The model has no upper limit on the largest molecule that can be produced in the chemistry but for the purposes of numerical simulation we assume that the largest molecule that can be produced is of size N. For the finite chemistry we exclude all the ligation reactions (and their reverse) that produce molecules of size larger than N. This results in $N-1$ coupled ordinary differential equations in $f=1$ case, given by:

$$\dot{x}_n = \sum_{i \le j, i+j=n} \left(k^F_{ij}x_ix_j - k^R_{ij}x_n\right) - \sum_{i=1, i\neq n}^{(N-n)} \left(k^F_{in}x_ix_n - k^R_{in}x_{i+n}\right) - 2\left(k^F_{nn}x_n^2 - k^R_{nn}x_{2n}\right) - \phi_n x_n \quad \text{(S1.4)}$$

for $n = 2, 3, \ldots, N$. Note that ∞ is replaced by $(N-n)$ in the second summation so that the largest molecule produced is of length N.

These ODEs were integrated using CVODE library of SUNDIALS package (Hindmarsh et al., 2005) and (for small N) XPPAUT (Ermentrout, 2002). The dependence of steady state concentrations on N is discussed separately in Supporting Appendix S2.

A. An example with $f = 1, N = 6$

The fully connected spontaneous chemistry has 9 reaction pairs of forward and reverse reactions given in the following table:

R1: A(1) + A(1) $\underset{k^R_{11}}{\overset{k^F_{11}}{\rightleftharpoons}}$ A(2)	R4: A(2) + A(2) $\underset{k^R_{22}}{\overset{k^F_{22}}{\rightleftharpoons}}$ A(4)	R7: A(1) + A(5) $\underset{k^R_{15}}{\overset{k^F_{15}}{\rightleftharpoons}}$ A(6)
R2: A(1) + A(2) $\underset{k^R_{12}}{\overset{k^F_{12}}{\rightleftharpoons}}$ A(3)	R5: A(1) + A(4) $\underset{k^R_{14}}{\overset{k^F_{14}}{\rightleftharpoons}}$ A(5)	R8: A(2) + A(4) $\underset{k^R_{24}}{\overset{k^F_{24}}{\rightleftharpoons}}$ A(6)
R3: A(1) + A(3) $\underset{k^R_{13}}{\overset{k^F_{13}}{\rightleftharpoons}}$ A(4)	R6: A(2) + A(3) $\underset{k^R_{23}}{\overset{k^F_{23}}{\rightleftharpoons}}$ A(5)	R9: A(3) + A(3) $\underset{k^R_{33}}{\overset{k^F_{33}}{\rightleftharpoons}}$ A(6)

From Eq. (S1.4), the 5 equations defining the dynamics are:

$$\begin{aligned}
\dot{x}_2 &= (k^F_{11}x_1^2 - k^R_{11}x_2) - (k^F_{12}x_1x_2 - k^R_{12}x_3) - 2(k^F_{22}x_2^2 - k^R_{22}x_4) - (k^F_{23}x_2x_3 - k^R_{23}x_5) - (k^F_{24}x_2x_4 - k^R_{24}x_6) \\
&\quad -\phi_2x_2, && \text{(S1.5a)} \\
\dot{x}_3 &= (k^F_{12}x_1x_2 - k^R_{12}x_3) - (k^F_{13}x_1x_3 - k^R_{13}x_4) - (k^F_{23}x_2x_3 - k^R_{23}x_5) - 2(k^F_{33}x_3^2 - k^R_{33}x_6) - \phi_3x_3, && \text{(S1.5b)} \\
\dot{x}_4 &= (k^F_{13}x_1x_3 - k^R_{13}x_4) + (k^F_{22}x_2^2 - k^R_{22}x_4) - (k^F_{14}x_1x_4 - k^R_{14}x_5) - (k^F_{24}x_2x_4 - k^R_{24}x_6) - \phi_4x_4, && \text{(S1.5c)} \\
\dot{x}_5 &= (k^F_{14}x_1x_4 - k^R_{14}x_5) + (k^F_{23}x_2x_3 - k^R_{23}x_5) - (k^F_{15}x_1x_5 - k^R_{15}x_6) - \phi_5x_5, && \text{(S1.5d)} \\
\dot{x}_6 &= (k^F_{15}x_1x_5 - k^R_{15}x_6) + (k^F_{24}x_2x_4 - k^R_{24}x_6) + (k^F_{33}x_3^2 - k^R_{33}x_6) - \phi_6x_6. && \text{(S1.5e)}
\end{aligned}$$

B. An example of inclusion of catalyst

Say reaction pairs R1 and R4 of the previous example are catalyzed by A(4) with catalytic strengths κ_4^{11} and κ_4^{22} respectively. Then the equations for x_2 and x_4 get modified to

$$\begin{aligned}
\dot{x}_2 &= (1 + \kappa_4^{11}x_4)(k^F_{11}x_1^2 - k^R_{11}x_2) - (k^F_{12}x_1x_2 - k^R_{12}x_3) - 2(1 + \kappa_4^{22}x_4)(k^F_{22}x_2^2 - k^R_{22}x_4) - (k^F_{23}x_2x_3 - k^R_{23}x_5) \\
&\quad -(k^F_{24}x_2x_4 - k^R_{24}x_6) - \phi_2x_2, && \text{(S1.6a)} \\
\dot{x}_4 &= (k^F_{13}x_1x_3 - k^R_{13}x_4) + (1 + \kappa_4^{22}x_4)(k^F_{22}x_2^2 - k^R_{22}x_4) - (k^F_{14}x_1x_4 - k^R_{14}x_5) - (k^F_{24}x_2x_4 - k^R_{24}x_6) \\
&\quad -\phi_4x_4. && \text{(S1.6b)}
\end{aligned}$$

The equations for $\dot{x}_3$, $\dot{x}_5$ and $\dot{x}_6$ remain as before. This chemistry, but with N extended to 15 is discussed in the context of bistability in Fig. 6 of main text. There all the spontaneous rate constants and x_1 have been chosen to be unity, all the ϕ_n's are equal and $\kappa_4^{11} = \kappa_4^{22} = \kappa$.

III. RATE EQUATIONS FOR A CHEMISTRY WITH $f = 2$

For a $f = 2$ chemistry, $\mathcal{F} = \{\mathrm{A}_1, \mathrm{A}_2\}$, a general molecule is given by $\mathrm{A} = (a_1, a_2)$, and the monomers are given by 2-tuples (1,0) and (0,1). There are $(n+1)$ molecules of length n, given by 2-tuples: $(n,0), (n-1,1), \ldots, (1,n-1), (0,n)$. A finite chemistry, *i.e.*, a chemistry in which the largest molecules that can be produced is of length N, has a total of $\frac{(N+1)(N+2)}{2} - 1$ molecules (including monomers), and hence $\frac{(N+1)(N+2)}{2} - 3$ rate equations.

A. An example with $f = 2$, $N = 3$

The fully connected spontaneous chemistry has 9 reaction pairs given in the following table:

R1: (0,1) + (0,1) $\rightleftharpoons$ (0,2)	R4: (0,1) + (0,2) $\rightleftharpoons$ (0,3)	R7: (0,1) + (2,0) $\rightleftharpoons$ (2,1)
R2: (0,1) + (1,0) $\rightleftharpoons$ (1,1)	R5: (0,1) + (1,1) $\rightleftharpoons$ (1,2)	R8: (1,0) + (1,1) $\rightleftharpoons$ (2,1)
R3: (1,0) + (1,0) $\rightleftharpoons$ (2,0)	R6: (1,0) + (0,2) $\rightleftharpoons$ (1,2)	R9: (1,0) + (2,0) $\rightleftharpoons$ (3,0)

Using Eq. (S1.1) suitably modified for a finite chemistry (as for $f = 1$ case in Section II), we can write the following rate equations:

$$\dot{x}_{(0,2)} = v_{\text{R1}} - v_{\text{R4}} - v_{\text{R6}} - \phi_{(0,2)}x_{(0,2)} \tag{S1.7a}$$

$$\dot{x}_{(1,1)} = v_{\text{R2}} - v_{\text{R5}} - v_{\text{R8}} - \phi_{(1,1)}x_{(1,1)} \tag{S1.7b}$$

$$\dot{x}_{(2,0)} = v_{\text{R3}} - v_{\text{R7}} - v_{\text{R9}} - \phi_{(2,0)}x_{(2,0)} \tag{S1.7c}$$

$$\dot{x}_{(0,3)} = v_{\text{R4}} - \phi_{(0,3)}x_{(0,3)} \tag{S1.7d}$$

$$\dot{x}_{(1,2)} = v_{\text{R5}} + v_{\text{R6}} - \phi_{(1,2)}x_{(1,2)} \tag{S1.7e}$$

$$\dot{x}_{(2,1)} = v_{\text{R7}} + v_{\text{R8}} - \phi_{(2,1)}x_{(2,1)} \tag{S1.7f}$$

$$\dot{x}_{(3,0)} = v_{\text{R9}} - \phi_{(3,0)}x_{(3,0)}. \tag{S1.7g}$$

$v_{\text{R}i}$ is the net forward flux of the reaction pair Ri (as given in the table above). Eqs. (S1.7) can be expanded as to get the equations in x's. For example, Eq. (S1.7b) becomes

$$\dot{x}_{(1,1)} = \left(k^F_{\text{R2}}x_{(0,1)}x_{(1,0)} - k^R_{\text{R2}}x_{(1,1)}\right) - \left(k^F_{\text{R5}}x_{(1,0)}x_{(1,1)} - k^R_{\text{R5}}x_{(2,1)}\right) - \left(k^F_{\text{R8}}x_{(0,1)}x_{(1,1)} - k^R_{\text{R8}}x_{(1,2)}\right) - \phi_{(1,1)}x_{(1,1)} \tag{S1.8}$$

where, $k^F_{\text{R}i}$ and $k^R_{\text{R}i}$ are the forward and reverse rate constant of reaction Ri, respectively.

When the catalysts are included, the reactions that involve catalysts are changed in the same way as in $f = 1$ case (see Section II.B of this appendix).

References

A. C. Hindmarsh, P. N. Brown, K. E. Grant, S. L. Lee, R. Serban, D. E. Shumaker, and C. S. Woodward, ACM Transactions on Mathematical Software **31**, 363 (2005), ISSN 00983500, URL `https://computation.llnl.gov/casc/sundials/main.html`.

B. Ermentrout, *Simulating, Analyzing, and Animating Dynamical Systems: A Guide to XPPAUT for Researchers and Students* (Society for Industrial Mathematics, Philadelphia, 2002), 1st ed., ISBN 0-89871-506-7, URL `http://www.math.pitt.edu/~bard/xpp/xpp.html`.
