# Supplementary material for: The Origin of Large Molecules in Primordial Autocatalytic Reaction Networks
Source: PLoS One. 2012 Jan 4;7(1):e29546. doi: 10.1371/journal.pone.0029546 (PMC3251582; doi:10.1371/journal.pone.0029546)
Supplement: Appendix S2 — -independence of results at large . (PDF) [file pone.0029546.s007.pdf]

The origin of large molecules in primordial autocatalytic reaction networks

Varun Giri and Sanjay Jain

Supporting Information: Appendix S2

N-independence of results at large N

While the chemistry considered in this model is an infinite one, numerical exploration requires us to work with a finite set of molecules that participate in the chemistry. This introduces an additional parameter N, the size of the largest molecule produced in the chemistry, in the model (for details see Appendix S1). Here we present evidence showing that certain important properties of the model become essentially N-independent at large enough N.

A. Uncatalyzed chemistry

The steady state concentrations for an uncatalyzed chemistry can be approximated by an exponential function, $x_n = ce^{-\gamma n} = c\Lambda^n$, where c and $\Lambda = e^{-\gamma}$ are constants (see Eq. (3) in the main text). We calculate Λ from the slope of the straight line fit on the plot of the log of the steady state concentrations, x_n, versus length, n. We found that for sufficiently large N, Λ becomes independent of N; see Fig. S2.1.

When $\Lambda < 1$, the concentrations of the large molecules are small. It is evident that if the concentrations of the large molecules produced in the chemistry are so small that their contribution to the dynamics of the (relatively) smaller molecules is negligible, then, including molecules larger than those already considered in such a chemistry would cause no difference to the results.

B. Catalyzed chemistry

For chemistries with catalyzed reactions, including ACSs, we find that for sufficiently large N, the numerical results are independent of N. In Fig. S2.2 we show how the steady state concentrations in the chemistry that includes ACS65 (defined by Eq. (5) in main text) depend upon N.

The results for the chemistry with $f = 2$ were also found to be N-independent (for sufficiently large values of N). We find that for $f = 2$ the N-independence is reached at much smaller values of N than for $f = 1$.

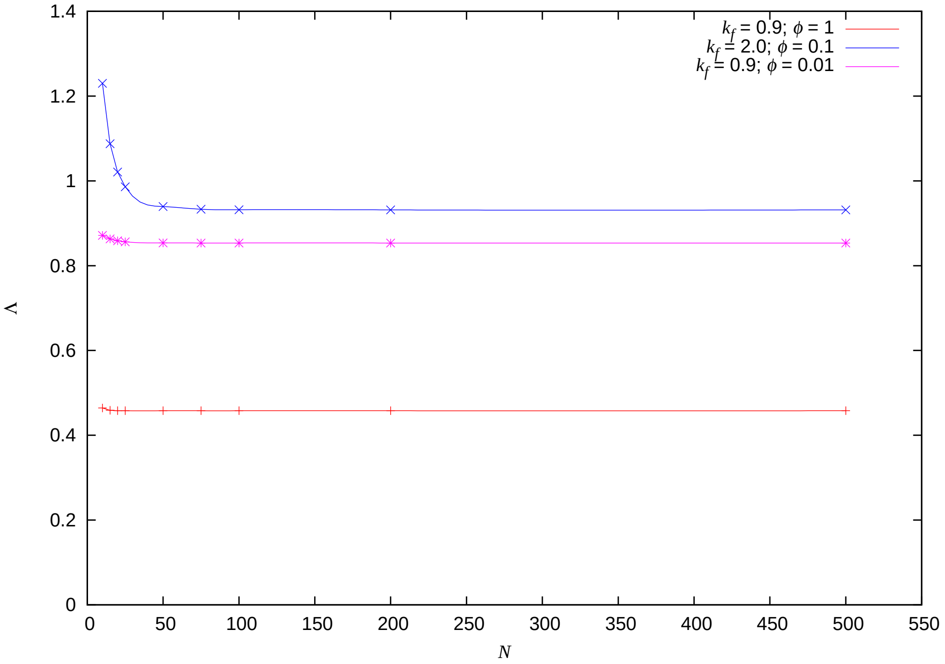

FIG. S2.1 **Dependence of Λ on N for uncatalyzed chemistries.** $A = k_r = 1$. We determine Λ at different values of N by fitting the steady state profiles to an exponential, $x_n = ce^{-\gamma n}$ (excluding the concentrations x_1 to x_4). We see a dependence of Λ on N for values of $N < 50$, but Λ becomes essentially independent of N for $N > 100$.

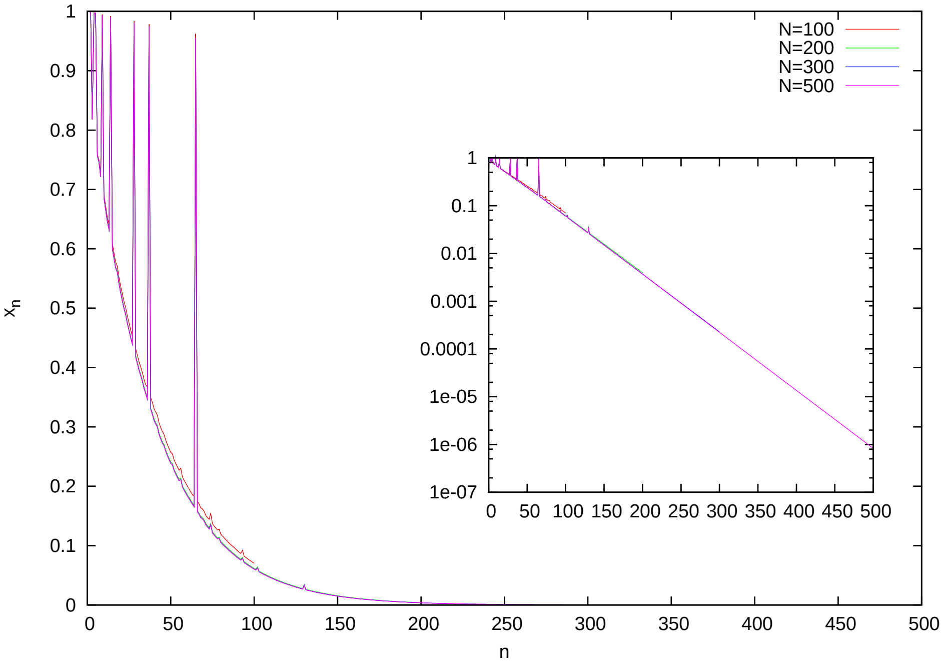

FIG. S2.2 **Dependence of steady state concentrations on N for a chemistry that includes an ACS.** The figure shows the steady state concentrations for the chemistry that includes ACS65 (Eq. 5 in main text) for $N = 100, 200, 300, 500$. In all cases $A = k_f = k_r = 1, \phi = 5$. The steady state concentrations show an N-dependence upto $N = 100$, but for $N \geq 200$ the profile becomes N-independent.
