# Supplementary material for: The Origin of Large Molecules in Primordial Autocatalytic Reaction Networks
Source: PLoS One. 2012 Jan 4;7(1):e29546. doi: 10.1371/journal.pone.0029546 (PMC3251582; doi:10.1371/journal.pone.0029546)
Supplement: Appendix S3 — Dimensionless rate equations and dependence of on dimensionless parameters. (PDF) [file pone.0029546.s008.pdf]

The origin of large molecules in primordial autocatalytic reaction networks

Varun Giri and Sanjay Jain

Supporting Information: Appendix S3

Dimensionless rate equations and dependence of Λ on dimensionless parameters

Eq. (2) in the main text can be cast in dimensionless form by introducing a concentration scale ω and a time scale τ. We discuss below the case of a homogeneous spontaneous chemistry.

Define dimensionless quantities $u_n \equiv x_n/\omega$, $t' \equiv t/\tau$, $k'_f \equiv k_f \omega\tau$, $k'_r \equiv k_r\tau$ and $\phi' \equiv \phi\tau$. Then

$$\dot{x}_n = \frac{dx_n}{dt} = \frac{d(u_n\omega)}{d(t'\tau)} = \frac{\omega}{\tau}\frac{du_n}{dt'}, \tag{S3.1}$$

and Eq. (2) in the main text becomes

$$\begin{aligned} \frac{\omega}{\tau}\frac{du_n}{dt'} &= \sum_{i\le j, i+j=n} \left(\frac{k'_f}{\omega\tau}(\omega u_i)(\omega u_j) - \frac{k'_r}{\tau}(\omega u_n) \right) - \sum_{i=1, i\neq n}^{\infty} \left(\frac{k'_f}{\omega\tau}(\omega u_i)(\omega u_n) - \frac{k'_r}{\tau}(\omega u_{i+n}) \right) \\ &\quad -2\left(\frac{k'_f}{\omega\tau}(\omega u_n)^2 - \frac{k'_r}{\tau}(\omega u_{2n}) \right) - \frac{\phi'}{\tau}(\omega u_n) \qquad \text{(S3.2)} \\ \frac{du_n}{dt'} &= \sum_{i\le j, i+j=n} \left(k'_f u_i u_j - k'_r u_n \right) - \sum_{i=1, i\neq n}^{\infty} \left(k'_f u_i u_n - k'_r u_{i+n} \right) - 2\left(k'_f u_n^2 - k'_r u_{2n} \right) - \phi' u_n \qquad \text{(S3.3)} \end{aligned}$$

Without loss of generality one can choose $\omega = x_1 = A$ and (whenever $k_r \neq 0$) $\tau = 1/k_r$. Then the dimensionless concentration variables satisfy the same equations as before, but with $A = k_r = 1$. There are now only two independent dimensionless parameters, k'_f and ϕ'. The dependence on all 4 parameters can be recovered at the end by replacing u_n by x_n/A, t' by tk_r, k'_f by $k_f A/k_r$ and ϕ' by ϕ/k_r. The behaviour of Λ as a function of k_f and ϕ (keeping $A = k_r = 1$) is shown in Fig. S3.1.

One may also choose $\tau = 1/\phi$ (whenever $\phi \neq 0$). In that case the two independent dimensionless parameters will be k'_f and k'_r. The behaviour of Λ as a function of k_f and k_r (keeping $A = \phi = 1$) is shown in Fig. S3.2.

When $k_r = 0$ and $\phi = 0$ both the above choices for scaling fail. In this case the system has no steady state for any finite N as $\dot{x}_N$ is always positive.

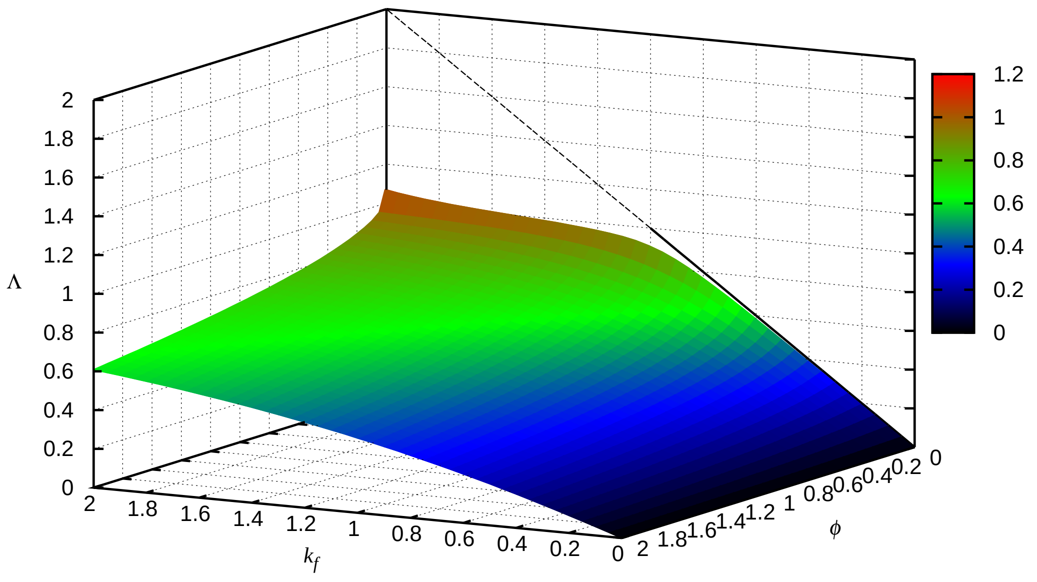

FIG. S3.1 **Behaviour of Λ as a function of k_f and ϕ.** The figure shows the dependence of Λ on k_f and ϕ for an uncatalyzed chemistry, keeping $A = k_r = 1$, $N = 100$. The curved surface was made with parameter values in the range $0 \leq k_f \leq 2$, $0.01 \leq \phi \leq 2$. Λ is found to be a monotonically increasing function of k_f and a monotonically decreasing function of ϕ. For $\phi = 0$, there is an analytical solution $\Lambda = k_f$ (see Eq. (4) in the main text). This was verified numerically in the range $0 \leq k_f \leq 1$ (see solid line at $\phi = 0$). In the region $k_f > 1$ the numerical integration does not converge at $\phi = 0$ as the steady state solution (Eq. (4) of the main text) $x_n = A\Lambda^{n-1} = Ak_f^{n-1}$ is numerically very large for large n. The dotted extension of the line ($1 < k_f \leq 2$) is simply the analytical result. Note that for most of the phase-space $\Lambda < 1$, except for very small values of ϕ.

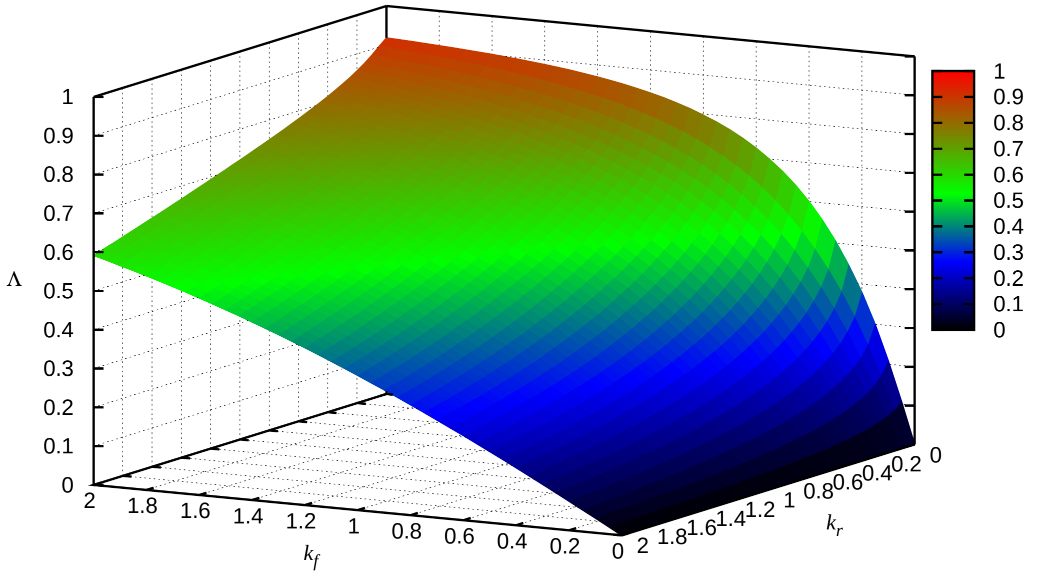

FIG. S3.2 **Behaviour of Λ as a function of k_f and k_r.** The figure shows the dependence of Λ on k_f and k_r for an uncatalyzed chemistry, keeping $A = \phi = 1$, $N = 100$. Λ is found to be a monotonically increasing function of k_f and a monotonically decreasing function of k_r.
